# Chromatin‐associated DEK proteins maintain H3K27me3 balance and coordinate developmental transitions in plants

**DOI:** 10.1111/nph.70704

**Published:** 2025-11-14

**Authors:** Miyuki Nakamura, Heinrich Bente, Maria Derkacheva, Matthew Gentry, Katarina Landberg, Mattias Thelander, Eva Sundberg, Lars Hennig, Claudia Köhler

**Affiliations:** ^1^ Department of Plant Biology, Uppsala BioCenter Swedish University of Agricultural Sciences and Linnean Center for Plant Biology SE‐75007 Uppsala Sweden; ^2^ Max Planck Institute for Molecular Plant Physiology Am Mühlenberg 1 14476 Potsdam Germany

**Keywords:** *Arabidopsis thaliana*, chromatin, DEK, histone H3K27me3, nucleosome binding‐protein

## Abstract

- Chromatin organization and histone modifications play essential roles in regulating gene expression during development. DEK is a conserved chromatin‐associated protein implicated in DNA topology and transcriptional regulation, yet its *in vivo* function in plants has remained elusive.
- To uncover *DEK* function, we used *Arabidopsis thaliana dek* mutants and performed genome‐wide analyses of histone modifications. Genetic and physical interactions with Polycomb Repressive Complex 2 (PRC2)‐associated proteins were examined, and transcriptome comparisons were conducted among single and multiple mutants.
- Loss of DEK function led to a genome‐wide increase in the PRC2‐mediated histone modification H3K27me3. DEK genetically and physically interacts with the PRC2‐associated protein LIKE HETEROCHROMATIN PROTEIN 1 (LHP1), and DEK deficiency partially restores H3K27me3 levels in the *lhp1* mutant. The *dek* multiple mutant exhibited enhanced H3K27me3 at PRC2 target genes and ectopic accumulation at pericentromeric interstitial telomeric repeats – similar to histone H1 mutants – suggesting altered chromatin accessibility. Combined *dek* and *lhp1* mutations intensified developmental defects and disrupted expression of many PRC2 target genes, including MADS‐box transcription factors. Transcriptome analyses revealed that DEK and chromatin remodeler alpha thalassemia/mental retardation syndrome X‐linked chromatin remodeler have opposing effects on gene expression.
- Our findings uncover DEK as a novel regulator of H3K27me3 homeostasis and chromatin structure, critical for coordinated plant development.

## Introduction

Gene expression is regulated not only by the presence or absence of transcription factors but also by higher order chromatin organization, established by histone modifications and variants, nucleosome compaction, and DNA topology. DEK is a chromatin‐bound protein with DNA‐binding motifs (Alexiadis *et al*., 2000; Waidmann *et al*., 2014). DEKs are potentially involved in multiple molecular processes, such as regulating gene expression and DNA repair (Privette Vinnedge *et al*., 2013). The *DEK* locus was initially found to be associated with acute myeloid leukemia (AML) in humans (von Lindern *et al*., 1992) and overexpression of *DEK* occurs in several tumor types of mammals ((Larramendy *et al*., 2002; Sanchez‐Carbayo *et al*., 2003; Grasemann *et al*., 2005; Carro *et al*., 2006; Abba *et al*., 2007) and reviewed in Riveiro‐Falkenbach & Soengas (2010)). Although the exact role of DEK proteins in chromatin regulation remains elusive, it has been suggested that DEKs preferentially bind to specific topological structures of DNA (e.g. negative supercoiled and cruciform) and histones *in vitro* (Alexiadis *et al*., 2000; Waldmann *et al*., 2002, 2003; Böhm *et al*., 2005). Human DEK interacts with the death domain‐associated protein (DAXX) (Hollenbach *et al*., 2002); however, a DAXX homolog is absent in the Arabidopsis genome. DAXX incorporates the histone variant H3.3, a marker for transcriptionally active chromatin, into gene‐rich regions (Drané *et al*., 2010; Lewis *et al*., 2010). It also marks both telomeres and pericentromeric heterochromatin in animals together with alpha thalassemia/mental retardation syndrome X‐linked chromatin remodeler (ATRX) proteins (Goldberg *et al*., 2010). Moreover, *Dek* knockdown causes ectopic histone H3.3 deposition in mouse cell lines (Ivanauskiene *et al*., 2014). Drosophila DEK interacts with Heterochromatin Protein 1α (HP1α), which binds to trimethylated lysine 9 of histone H3 (H3K9me3) (Kappes *et al*., 2011). Drosophila *Dek* knockdown causes abnormalities in heterochromatin silencing. A recent study revealed the cryo‐EM structure of the human DEK protein and showed that the DEK–nucleosome interaction mediates linker DNA reorientation and induces chromatin compaction (Kujirai *et al*., 2025). Together, these observations suggest that DEK is required for general chromatin organization in animals.

Previous work from our group identified DEK3 and DEK4 to co‐immunoprecipitate with LIKE HETEROCHROMATIN PROTEIN 1 (LHP1), but the functional relevance of this interaction remained unknown (Derkacheva *et al*., 2016). LHP1 is a plant‐specific homolog of the animal HP1 (Gaudin *et al*., 2001). Unlike animal HP1, which is associated with H3K9me3, LHP1 is responsible for maintaining trimethylation on lysine 27 of histone H3 (H3K27me3) (Turck *et al*., 2007; Zhang *et al*., 2007; Exner *et al*., 2009). The Arabidopsis genome contains four homologous *DEK* genes: *DEK‐LIKE1*, *DEK‐LIKE2*, *DEK3*, and *DEK4*. The Arabidopsis DEK proteins were previously published as AtDEK‐1, AtDEK‐2, AtDEK‐3, and AtDEK‐4 (Pendle *et al*., 2005). However, since the gene name *DEK1* was originally published as *DEK1(DEFECTIVE KERNEL 1)* in maize (Lid *et al*., 2002), the naming of *AtDEK‐1* in Arabidopsis as *AtDEK1* is ambiguous. To avoid further confusion, here we renamed *DEK* homolog *AtDEK‐1* and *AtDEK‐2* as *DEK‐LIKE1* (*DEKL1*) and *DEK‐LIKE2* (*DEKL2*), respectively. DEK3, encoded by one of the homologs, functions as a chromatin‐bound protein (Waidmann *et al*., 2014). It has been suggested that DEK3 mediates stress responses (Waidmann *et al*., 2014; Brestovitsky *et al*., 2019). DEK3 and its close homolog DEK4 bind to and facilitate RNA polymerase II accumulation at some loci of the *FLOWERING LOCUS C* (*FLC*) and *MADS AFFECTING FLOWERING* (*MAF*) family, encoding flowering repressors in response to cold temperature (Zong *et al*., 2021). Loss of function of *DEK3* affects histone H2A.Z distribution (Brestovitsky *et al*., 2019), likely affecting the expression of genes such as *FLC* and *MAFs*. DEK3 was shown to physically interact with histones H3, H4, H2A.Z, and topoisomerase Iα as well as members of the cohesion complex (Waidmann *et al*., 2014; Brestovitsky *et al*., 2019; Sundaram *et al*., 2024). Thus, DEKs are involved in epigenetic regulation at different levels; however, there is limited mechanistic understanding of the functional role of DEK *in vivo*.

In this study, we unveiled the comprehensive scope of plant DEKs by producing the multiple mutant of *DEK* in Arabidopsis and the knockout (KO) mutant in moss. We found that defects in DEKs had a significant impact on the state of H3K27me3. The H3K27me3 modification is catalyzed by the Polycomb Repressive Complex 2 (PRC2) and is important for silencing of key developmental regulatory genes in both animals and plants. Defects in LHP1 function cause misexpression of many PRC2 target genes and genome‐wide alterations of H3K27me3 levels (Veluchamy *et al*., 2016; Wang *et al*., 2016). Our investigation revealed that the multiple mutant *lhp1;dek* strongly enhanced the *lhp1* morphological phenotype. Interestingly, however, *lhp1;dek* exhibited intermediate levels of H3K27me3 between *dek* and *lhp1*, but enhanced gene deregulation, in particular of PRC2‐target genes. In addition to increased H3K27me3 in PRC2‐target genes, we also found a strong gain of H3K27me3 at large pericentromeric interstitial telomeric repeat (ITR) domains, resembling a phenotype observed in mutants depleted for histone H1. Together, our study reveals DEK as an evolutionarily conserved regulator of H3K27me3 homeostasis and chromatin structure, critical for coordinated plant development.

## Materials and Methods

### Plant material and growth conditions

In this study, the *Arabidopsis thaliana* accession Columbia‐0 and previously published mutant alleles of *lhp1‐6* (Exner *et al*., 2009), *dekl1‐2* (SALK_113239), *dekl2‐3* (SALK_137152C), *dek3‐3* (GK_179G02), *dek4‐2* (SALK_060488C), and *atrx‐3* (SALK_024609) were used. PCR for genotyping was performed with gene‐specific primers (Supporting Information Table S1). Plants were grown on soil under long‐day (LD) conditions: 16 h (h) : 8 h, light : dark or short‐day (SD) conditions: 8 h : 16 h, light : dark at 22°C ± 2. Sterilized seeds were kept at 4°C for 2 d in the dark before moving to 22°C under light conditions. Plants were germinated and grown for the first 10–12 d in ½ Murashige & Skoog (MS) plates containing MS salt (Murashige & Skoog, 1962), 1% sucrose, 0.1% MES (2‐(N‐morpholino)ethanesulfonic acid), and 0.8% agarose. For the expression assays, whole seedlings were harvested 30–60 min before darkness. For determining Arabidopsis flower numbers until terminal flower formation, the total number of flowers was counted from 7‐w‐old and 8‐wk‐old plants under SD and LD conditions, respectively. The 3^rd^–5^th^ leaves were harvested from 5‐w‐old plants grown under SD conditions. Leaves were scanned and measured by ImageJ (https://imagej.nih.gov/ij/).

For experiments involving moss, the *Physcomitrium* (*Physcomitrella*) *patens* ecotype Reute was used (Hiss *et al*., 2017). For subcultivation, protonemal tissue was blended and grown on cellophane‐covered minimal medium plates containing BCD medium (Thelander *et al*., 2007) supplemented with 5 mM ammonium tartrate and 0.8% agar at 25°C under constant white light from fluorescent tubes (FL40SS W/37; Toshiba, Yokosuka, Japan) at 30 μmol m^−2^ s^−1^ in a Sanyo MLR‐350 light chamber with side irradiation. For phenotypic analysis, all experiments were repeated at least twice. Protonema was subcultured three times and then shaped into small balls (1–2 mm in diameter), inoculated on BCD plates, and grown under the same conditions as described previously. Colonies were analyzed, and images were taken at indicated time points using a Leica M205FA stereo microscope and LAS AF software (Leica Microsystems, Wetzlar, Germany).

### Bimolecular fluorescence complementation analyses in *Nicotiana benthamiana* leaves

The *DEKL2* (AT5G63550) and *DEK4* (AT5G55660) coding sequences (cDNA) were cloned into the vector pSITE‐nYFP‐N1 (Martin *et al*., 2009). The pSITE‐nYFP‐N1‐2b construct was a gift from Dr E. Savenkov (SLU, Uppsala Biocenter). The LHP1 cDNA was cloned into the vector pSITE‐cYFP‐C1 (Martin *et al*., 2009), and the H3.3 cDNA was cloned into pUBC‐CFP (Grefen *et al*., 2010). The *DEKL2* clone was obtained from Arabidopsis Biological Resource Center (ABRC: abrc.osu.edu). Suspensions of *Agrobacterium tumefaciens* (strain GV3101) were infiltrated into the leaves of *Nicotiana benthamiana* as previously described. At 48 h after infiltration, water‐mounted sections of leaf tissue were examined by confocal microscopy (Zeiss LSM 710). The yellow fluorescent protein (YFP) channel was excited with a 514‐nm laser, the emission signal was collected in a wavelength window between 520 and 582 nm. The cyan‐fluorescent protein (CFP) channel was excited with a 458‐nm laser, the emission signal was collected in a wavelength window between 456 and 514 nm. The Cucumber mosaic virus 2b protein localizes to the nucleus (Wang *et al*., 2004) and served as a negative control in this assay. H3.3‐CFP served as a marker for the nucleus.

### Sequence alignment and phylogenetic analysis

In addition to four DEK proteins from Arabidopsis – DEKL1 (AT3G48710), DEKL2 (AT5G63550), DEK3 (AT4G26630), and DEK4 (AT5G55660) – we used the following amino acid sequences for the phylogenetic analysis: *Capsella rubella* Reut.006282447, (XP_006282447, XP_023636051, and XP_023633277), *Populus trichocarpa* Torr. and A.Gray ex Hook. (0012s10420g, 0015s11280g, 0001s37610g, and 0011s09560g), *Oryza sativa* L. (Os03g0596900 and Os01g0924900), *Amborella trichopoda* Baill. (XP_011627798.1), *Picea abies* (L.) H. Karst. (MA_108165g0010, MA_20536g0010, MA_9561633g0010, and MA_8963515g0010), *Selaginella moellendorffii* Hieron. (XP_002963625), *Physcomitrium patens* (Hedw.) Bruch and Schimp. (PNR40634.1/Pp3c14_4960V3.1), *Homo sapiens* L. (NP_003463.1), *Mus musculus* L. (NP_080176.2), and *Drosophila melanogaster* Meigen (NP_652058.1). For sequences conservation analysis between Arabidopsis and animals, amino acid sequences were aligned by T‐Coffee (Notredame *et al*., 2000) (http://tcoffee.crg.cat) and drawn with ESPript 3 (Robert & Gouet, 2014).

For phylogenetic analysis, amino acid sequences of DEK homologs were identified in Phytozome 12 (https://phytozome.jgi.doe.gov/pz/portal.html), NCBI (https://blast.ncbi.nlm.nih.gov), and Congenie (http://congenie.org/) using Arabidopsis DEKL1 to DEK4 as queries. Full‐length or whole available amino acid sequences of representative organisms were aligned with the ClustalW algorithm in the Mega7 software (Kumar *et al*., 2016). The phylogenetic tree was drawn using the neighbor‐joining method with 500 bootstrap replicates (Saitou & Nei, 1987). In this analysis, the amino acid sequence of another Arabidopsis DEK_C protein AT3G19080 was applied as an outgroup.

### Generation of *P. patens* knockout constructs and lines

To produce the *PpDEKL* G418R KO construct (Fig. S1a), a 1014‐bp *PpDEKL* 5′ flanking ragment was first amplified with primers PpDEKL‐5′‐F and PpDEKL‐5′‐R, trimmed to 998 bp with *Bam*HI, and ligated into *Bam*HI sites of plasmid pMT164 (Thelander *et al*., 2007). A 1002‐bp *PpDEKL1* 3′ flanking fragment was next amplified with primers PpDEKL‐3′‐F and PpDEKL‐3′‐R, trimmed to 986 bp with *Hpa*I, and ligated into *Hpa*I sites of the product of the first ligation. The resulting construct was linearized with *Xba*I/*Asc*I before transformation. Protoplast transformation and selection of stable transformants in the presence of 50 μg ml^−1^ G418 (11 811 023; Thermo Fisher Scientific, Waltham, MA, USA) was carried out as previously described (Schaefer *et al*., 1991). Stably transformed lines were checked for correct construct integration by PCRs using genomic DNA as a template to confirm 5′‐ and 3′‐junctions, as well as PCRs using either genomic DNA or cDNA as templates to confirm the loss of internal gene sequences (Fig. S1b,c).

### RNA isolation and RT‐qPCR

Total RNA was extracted from 7‐day‐old or 10‐day‐old seedlings of each genotype by RNeasy Plant mini kit (Qiagen, Germany #74904). After DNase I (Thermo Fisher Scientific, Waltham, MA, USA) treatment, the reverse transcription reaction was performed with oligo(dT) and random hexamer primers using the RevertAid First Strand cDNA synthesis kit (Thermo Scientific). Quantitative polymerase chain reaction with gene‐specific primers (Table S1) was performed using a MyiQ Single Color Real Time PCR detection system (BIO‐RAD, CA, USA) and the HOT FIREPol® EvaGreen® qPCR Mix Plus (Solis Biodyne, Tartu, Estonia) according to the manufacturer's instructions. Gene expression values are calculated relative to a *PP2a* reference gene (AT1G13320). The primers used in this study are listed in Table S1. Each genotype background has three biological replicates.

### RNA‐seq and data analysis

Poly(A) tail RNA was enriched from total RNA using NEBNext Poly(A) mRNA Magnetic Isolation Module (NEB #E7490, MA, USA). RNA‐seq libraries were prepared with NEBNext® Ultra II Directional RNA Library Prep Kit for Illumina (NEB #E7709) according to the manufacturer's manuals. Barcoded libraries with NEBNext Multiplex Oligos for Illumina (NEB #E7760) were sequenced in Novaseq 6000 as 150‐bp paired‐end in Novogene (Hongkong, China). Each genotype background has three biological replicates except the *lhp1;dek* background, which has two biological replicates. Sequence data were filtered for quality control using FASTX‐toolkit with ‐q 20 ‐p 80 ‐Q 33 option, ensuring at least 80% of its bases with > 99% base call accuracy in each read. After adaptor trimming using cutadapt (Martin, 2011), read1 and read2 files were paired using the BBmap software and were mapped by Hisat2 (v.2.1.0) with ‘‐k 3’ option to the Arabidopsis reference genome TAIR10 (sourceforge.net/projects/bbmap/; Kim *et al*., 2019). Aligned reads were counted in a strand‐specific manner using HTseq (v.0.9.1) over Arabidopsis_thaliana.TAIR10.45.gtf (Anders *et al*., 2015). Differentially expressed genes (DEGs) were identified by the edgeR package in R (v3.6.3) (Robinson *et al*., 2010). The threshold of DEGs is false discovery rate (FDR) < 0.01 and | log_2_ Fold change | > 1. Principal component analysis (PCA) was performed on log‐transformed read counts for each gene by the *prcomp* function of the stats package in R. Enrichment of gene ontology was performed with g:profiler (Reimand *et al*., 2007; Kolberg *et al*., 2023) and representative Gene Ontology terms were selected using REVIGO (Supek *et al*., 2011). Fold enrichment was calculated as follows: Fold Enrichment = {(Overlap/the number of query genes)}/{(the number of genes in the term / the total count of unique genes in the category)}. The Heatmap of developmental expressions and gene subset with high loading scores on the second PC (PC2) were drawn with the heatmap.2 function of the gplot package in R using the AtGenExpress dataset (Schmid *et al*., 2005).

### Chromatin immunoprecipitation (ChIP) and high‐throughput sequencing

Three hundred milligrams of 12‐day‐old seedlings was cross‐linked in 1% formaldehyde solution. Chromatin isolation was performed as previously described (Luo & Lam, 2014). Isolated chromatin was sonicated using Covaris S2 Focused‐ultrasonicator (duration: 20 min, duty cycle: 5%, intensity: 4, cycles per burst: 200) and then incubated with each antibody at 4°C with rotation overnight using anti‐H3K27me3 (#07‐449; Millipore, MA,USA), and antihistone H3 (ab1791; Abcam, Cambridge, UK) antibodies. Moreover, Protein G Dynabeads (Thermo Fisher) were added and incubated at 4°C with rotation for 2 h. Subsequently, Beads‐Ab‐chromatin were washed using the following buffers: low salt buffer (150 mM NaCl, 0.1% SDS, 1% Triton X‐100, 1 mM EDTA, 50 mM Tris–HCl, 0.1% sodium deoxycholate, complete EDTA‐free Protease Inhibitor Cocktail (Sigma‐Aldrich, MO, USA); pH 7.8) twice, high salt buffer (the same composition as low salt buffer except 500 mM NaCl) twice, once with 1 ml of LiCl buffer (0.25 M LiCl, 1 mM EDTA, 10 mM Tris–HCl,1% IGEPAL CA‐630, 1% sodium deoxycholate, pH 7.8) once, and 1× TE once. Precipitated chromatin was decross‐linked using elution buffer (10 mM Tris–HCl, pH 7.8, 0.3 M NaCl, 5 mM EDTA, and 0.5% SDS) at 65°C overnight. DNA was extracted with Monarch PCR & DNA Cleanup Kit (NEB) after RNase A (Nippon Gene, Tokyo, Japan) and Proteinase K (Thermo Fisher) digestion for 2 h at 37°C, and subsequently was precipitated with ethanol. Recovered DNA amounts were estimated using Qubit dsDNA High Sensitivity Assay kit (Thermo Fisher). The libraries were prepared using ThruPLEX DNA‐seq kit (Takara, Shiga, Japan) and DNA HT Dual Index kit (Takara) following the manufacturer's instructions. Paired‐end reads of 150 bp were sequenced with an Illumina NextSeq 1000. Two biological replicates were conducted independently.

### Data processing for ChIP‐seq

After quality trimming, the first 80 nucleotides from the forward reads were aligned to the TAIR10 reference genome using Bowtie2 with the ‘best’ option. The peaks of histone modifications were called using Macs3 (v.3.0.0a7; https://github.com/macs3‐project/MACS) (Zhang *et al*., 2008) with the following options: ‘‐g 1.3e8 ‐B ‐‐nomodel ‐‐extsize 147 ‐q 0.01’. The *q*‐value threshold of 0.01 corresponds to a 1% FDR for peak detection. The alignment data of anti‐H3 chromatin immunoprecipitation followed by sequencing (ChIP‐seq) from each corresponding genotype background were used as a control. The idr2d package in R was used to test consistency between replications of the ChIP peaks (Li *et al*., 2011). Irreproducible discovery rate (IDR) assesses reproducibility between biological replicates, with values < 0.05 indicating high reproducibility. Peaks with IDR < 0.05 were extracted as reproducible peaks. The lack of spike‐in controls represents a limitation for absolute quantification, although the comparative analysis of modification patterns between genotypes remains robust due to consistent multiple biological replicates and normalization to H3 as an internal control. The analysis focused on relative changes in peak patterns rather than absolute modification levels. H3K27me3 signal peak was visualized using the Integrative Genomics Viewer (IGV) (Robinson *et al*., 2011). Metagene profiles of the H3K27me signals around CURLY LEAF (CLF)‐ and SWINGER (SWN)‐binding sites were generated using computeMatrix and plotProfile from the Deeptools (Ramirez‐Prado *et al*., 2019). The dataset of the CLF‐ and SWN‐binding sites was obtained from NCBI Gene Expression Omnibus GSE108960 (Shu *et al*., 2019). To identify regions with H3K27me3 gain or loss across the whole genome, genomic regions were segmented into 1‐kb bins using a 500‐bp sliding window. For each bin, read counts were normalized by the total number of reads in the corresponding sample. Histone modification signals were calculated as the mean of normalized read counts from two biological replicates. To compare between genotypes, bins showing a signal difference greater than 0.5 were classified as gain bins, while those with a difference < −0.5 were classified as bins with a signal loss. ChIP‐seq data for H3K27me3 in the *h1* double mutant and the corresponding wild‐type (WT) control were retrieved from GSE160410. H3K27me3 signals in *trb123* were retrieved from Wang *et al*. (2023).

### Sequence analysis of pericentromeric repeat regions

To analyze repeat sequences, the primary DNA sequences corresponding to ectopic H3K27me3 peaks observed in dek were annotated using the Tandem Repeat Annotation and Structural Hierarchy (TRASH) tool (Wlodzimierz *et al*., 2023). For visualization in IGV, the most frequent sequence, TAGGGTT, was used as a query in the bedtools nuc function to quantify its occurrence within 200‐bp genomic bins (Quinlan & Hall, 2010).

## Results

### Defects in *DEKs* promote the transition to reproduction in both *Arabidopsis* and *Physcomitrium*

**Fig. 1:**
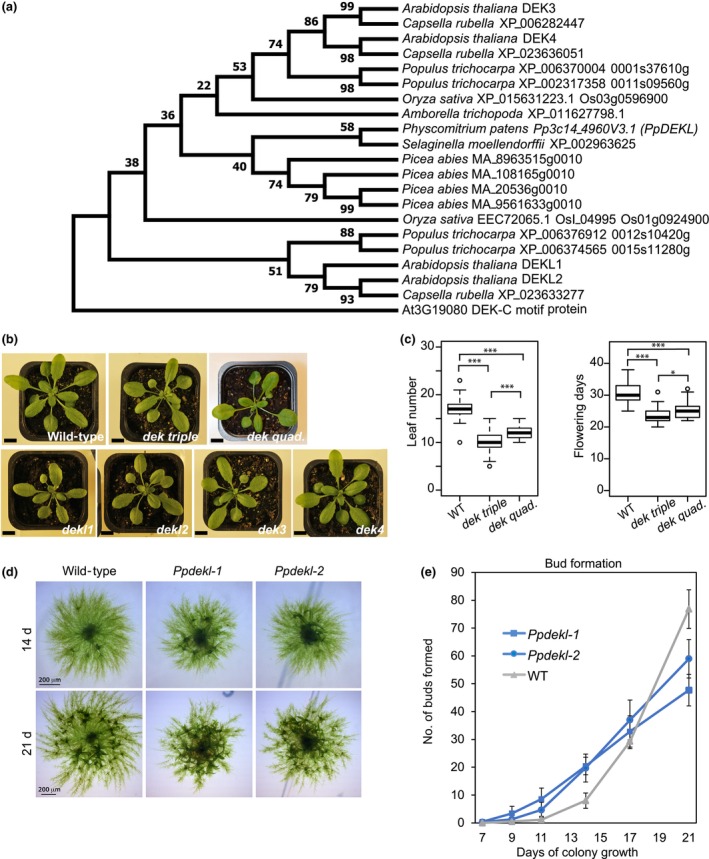
Defects in *DEK* genes influence phase transition in both *Arabidopsis* and *Physcomitrium*. (a) Phylogenetic tree was built using full‐length or available sequences of DEK homologs from representative plants. AT3G19080 containing DEK_C domain was used as an outgroup. Bootstrap values are shown in percentages at nodes. Sequence accession numbers from Phytozome 12, NCBI, and Congenie are shown at terminal branches. (b) *dek* single, triple, and quadruple mutants are morphologically similar to wild‐type (3 w old in long‐day (LD) condition). Bars, 10 mm. (c) Flowering time of the *dek* triple and quadruple mutants measured by leaf number (left panel) and days to flowering (right panel) under LD conditions. Box plots display median (center line), first and third quartiles (box edges), and range within 1.5× interquartile range (whiskers). Outliers are shown as individual points. Asterisks indicate significant differences (one‐way ANOVA followed by Tukey test). *, *P* < 0.01; ***, *P* < 0.0001. (d) Morphology of *Physcomitrium* protonema colonies after 14 and 21 d of growth. (e) Total number of buds initiated per colony between 7 and 21 d of growth. Bars show SD.

DEK proteins have two conserved motifs, DEK_C and the scaffold attachment factor (SAF) box, both of which are DNA‐binding motifs (Kipp *et al*., 2000). These motifs are conserved between animals and Arabidopsis (Fig. S2). A BLAST search using Arabidopsis DEK as a query identified DEK homologs in other plant species. Mosses, ferns, and the early diverged angiosperm *A. trichopoda* have only one DEK homolog, similar to animal species. By contrast, the gymnosperm *P. abies* has four DEK homologs (Fig. 1a). All nonangiosperm homologs in plants belong to the same clade as Arabidopsis DEK3 and DEK4, while Arabidopsis DEKL1 and DEKL2 belong to an angiosperm‐specific clade.

To investigate the functional roles of *DEKs in vivo*, we isolated and characterized T‐DNA insertion mutants for each *DEK* gene (Fig. S3a). Mutants *dekl1‐2, dekl2‐3*, and *dek3‐3* lacked detectable full‐length transcripts, while the *dek4‐2* mutant showed very weak transcript signals for full‐length *DEK4* (Fig. S3b). Thus, *dekl1‐1*, *dekl2‐1*, and *dek3‐1* are potentially severe loss‐of‐function alleles, and *dek4‐1* is a potential knockdown. Single mutants for each *DEK* gene showed no apparent morphological abnormalities (Fig. 1b). A recent study reported that the *dek3 dek4* double mutant shows a weak early‐flowering phenotype (Zong *et al*., 2021), revealing functional redundancy between *DEK* genes. To test whether DEK3 and DEK4 act functionally redundant with DEKL1 and DEKL2, we generated multiple higher order *dek* mutants. Similar to previously reported for the *dek3 dek4* double mutant (Zong *et al*., 2021), the *dekl2 dek3 dek4* triple mutant and the *dekl1 dekl2 dek3 dek4* quadruple mutant were slightly earlier flowering (Fig. 1c).

To address DEK function in a genetic system with reduced genetic redundancy, we knocked out the single *DEK* gene (Pp3c14_4960V3.1) in the moss *P. patens*. Again, to distinguish this gene from moss *DEFECTIVE KERNEL1* (*PpDEK1*) (Perroud *et al*., 2014), we named this gene *PpDEK_LIKE* (*PpDEKL*). We generated two independent knock out (KO) mutants that contained large deletions in the *PpDEKL* coding region (Fig. S1). Both mutants showed identical phenotypes; specifically, the colony diameter was reduced, and bud emergence occurred earlier in young protonemal colonies than in wild‐type (WT) (Fig. 1d,e). Statistical comparisons at each time point using the Wilcoxon test (with adjustment for multiple comparisons) revealed significant differences beginning on Day 11. At Day 21, the WT  plants had significantly more buds than the KO lines (adjusted *P*‐values; 7‐d: 0.174, 9‐d: 0.075, 11‐d: 0.007, 14‐d: 0.002, 17‐d: 0.075, and 21‐d: 0.002). In older colonies of *ppdekl* mutants, the total number of buds was lower than in WT, probably due to the difference in colony size (Fig. 1d,e). Interestingly, also *Pplhp1* KO plants show precocious gametophore bud formation (Parihar *et al*., 2019), similar to *Ppdekl* KO plants. Taken together, in both *Arabidopsis* and *Physcomitrium*, deficiencies in DEK function cause premature phase transitions on the route toward reproduction, revealing a conserved role of DEKs in timing the onset of reproduction.

### DEKs colocalize with LHP1 in plant nuclei

Previous work from our group showed that DEK3 and DEK4 co‐immunoprecipitated with LHP1 (Derkacheva *et al*., 2016). *DEK* genes and *LHP1* are highly expressed in the shoot apex and early flower stages in Arabidopsis (Fig. S4a) (Schmid *et al*., 2005). To validate the colocalization between DEKs and LHP1, we conducted bimolecular fluorescence complementation assays. Since DEKL1 and DEKL2 as well as DEK3 and DEK4 are highly similar to each other, we selected DEKL2 and DEK4 to test interactions with LHP1. We transiently co‐expressed DEKL2 fused to the N‐terminal region of the YFP (nYFP) and DEK4 fused to nYFP together with LHP1 fused to the C‐terminal region of YFP (cYFP) in *N. benthamiana* leaves and monitored YFP signals in nuclei expressing the respective proteins (Fig. S4b). Transformed cells were identified by co‐expressing H3.3 fused to the CFP. Cells expressing DEKL2‐nYFP and DEK4‐nYFP together with LHP1‐cYFP showed a clear fluorescent signal in nuclei, while the expression of a cucumber mosaic virus nuclear 2b protein (Wang *et al*., 2004), used as a negative control, fused to nYFP, did not show fluorescence when co‐expressed with LHP1‐cYFP. These results confirm that DEKL2 and DEK4 physically interact with LHP1 in the nucleus.

### Synergistic morphological defects of *dek* mutants in combination with *lhp1*

**Fig. 2:**
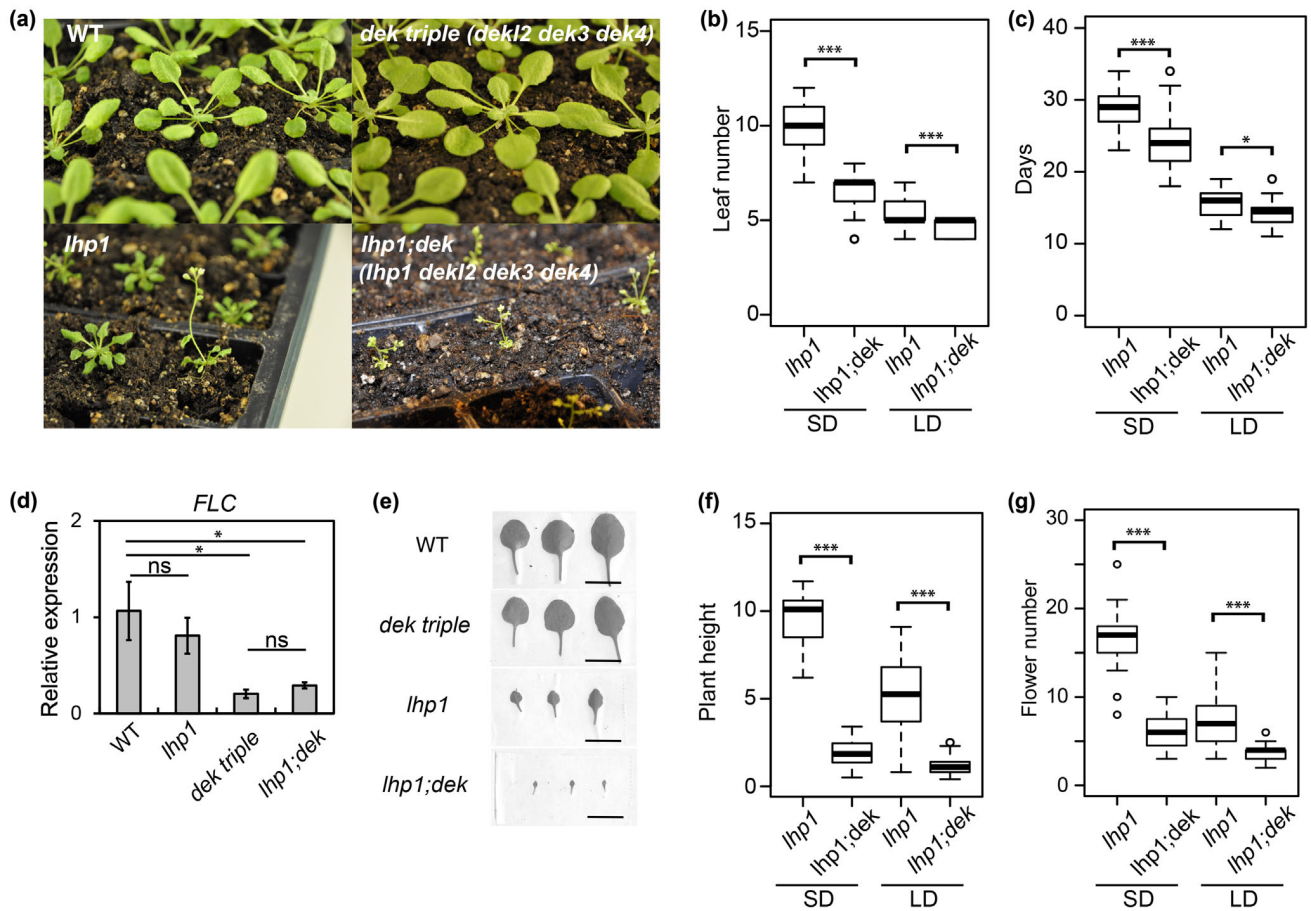
*dek* mutations enhance the *lhp1* abnormal phenotypes. (a) Phenotype of 5‐w‐old plants under short‐day (SD) conditions. Most of the *lhp1;dek* mutant plants flowered at this stage. (b, c) Flowering time of the *dek* triple and quadruple mutants was earlier both considering leaf number (b) and days to flowering (c) under SD and long‐day (LD) conditions. Asterisks indicate significant differences (Student's *t*‐test). *, *P* < 0.05; ***, *P* < 0.0005. (d) *FLOWERING LOCUS C* expression by reverse transcription quantitative polymerase chain reaction in wild‐type, *lhp1*, *dek* triple, and *dek;lhp1* quadruple mutants. ns, not significant; *, *P* < 0.05. (e–g) *dek* enhanced phenotypes of *lhp1*. Leaf size of the 3^rd^–5^th^ leaf in 5‐wk‐old plants under SD (e). Plant height in *lhp1;dek* was shorter than *lhp1* (f). *lhp1;dek* produced fewer flowers than *lhp1* (g). Bars, 1 cm. Asterisks indicate significant differences (Student's *t*‐test). ***, *P* < 0.0005.

Since we identified DEKs as potential interactors of LHP1, we investigated the genetic interaction between mutants in *DEK* and *LHP1* genes. We generated a *lhp1 dekl2 dek3 dek4* (hereafter, *lhp1;dek*) quadruple mutant, resulting in more severe phenotypes than *lhp1* (Fig. 2a). The *lhp1;dek* quadruple mutant flowered significantly earlier under both LD and SD conditions than *lhp1*, with a pronounced shortening of the time to flower under SD conditions (Fig. 2b,c). Early flowering correlated with reduced *FLC* expression in the *dek* triple mutant, which was not further reduced in the *lhp1;dek* mutant (Fig. 2d). The presence of *dek* mutations strongly enhanced the *lhp1* morphological phenotype, such as short plant height, reduced flower numbers, and small leaf size (Fig. 2e–g).

Since the *dekl2 dek3 dek4* triple and *dekl1 dekl2 dek3 dek4* quadruple mutants (hereafter, referred to as *dek*) were phenotypically nearly indistinguishable from WT (Fig. 1b), the enhanced phenotypes of the *lhp1;dek* quadruple mutant were striking. We failed to obtain the quintuple mutant *lhp1 dekl1 dekl2 dek3 dek4* among the progeny of *dekl1/+;dekl2/−;dek3/+;dek4/−;lhp1/+*, suggesting that the combination of *lhp1* and *dek* affects fertility. Together, the genetic interaction between *LHP1* and *DEKs* suggests that both proteins cooperatively impact plant development.

### Loss of DEK function causes increased H3K27me3 *in vivo*

**Fig. 3:**
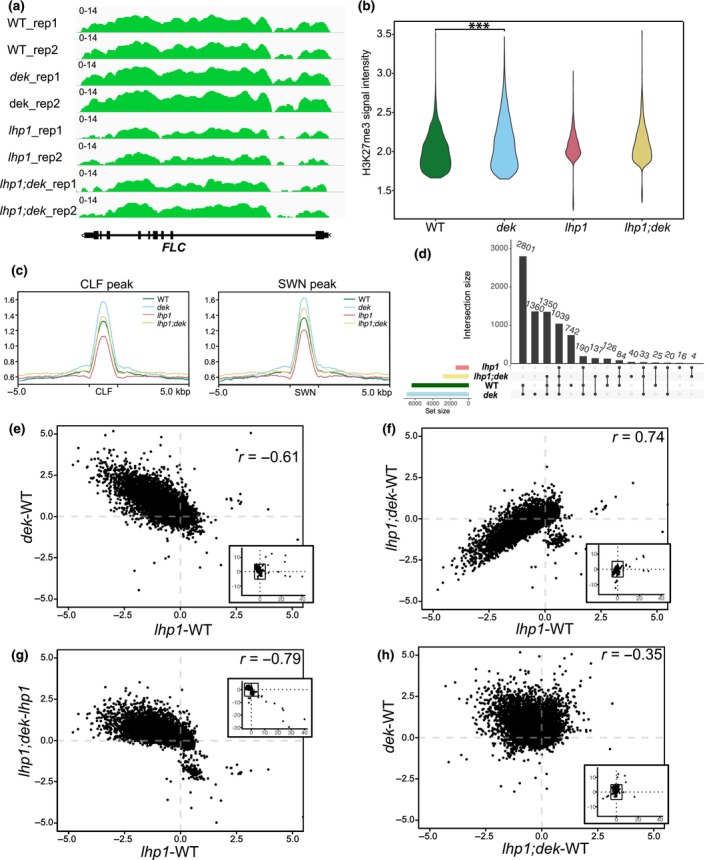
H3K27me3 abundance is increased in *dek*. (a) IGV genome browser screenshots showing the accumulation of H3K27me3 at the *FLOWERING LOCUS C (FLC)*. (b) Distribution of H3K27me3 fold changes against input in called peaks of each genotype. ***, *P* < 0.0005. (c) H3K27me3 signals at CURLY LEAF (CLF)‐ and SWINGER (SWN)‐binding sites and the flanking regions ±5 kbp. (d) Upset plot for genes with H3K27me3 peaks. (e–h) Correlation of H3K27me3 changes in each mutant. Each dot corresponds to one gene. Normalized read counts overlapping each gene and adjacent regions were summed up and averaged between two biological replicates. The main plot excludes outliers; the inset displays all data. H3K27me3 difference (d) in *lhp1* compared to wild‐type (WT) vs *dek* compared to WT, (e) in *lhp1* compared to WT vs in *lhp1;dek* compared to *lhp1*, (f) *lhp1;dek* compared to WT vs in *dek* compared to WT, and (g) *lhp1* compared to WT vs in *lhp1;dek* compared to *lhp1*.

To test whether the strong morphological abnormalities of *lhp1;dek* were caused by chromatin alterations, we performed ChIP‐seq of the H3K27me3 modification, which is known to be reduced in *lhp1* (Veluchamy *et al*., 2016). We observed that the *dek* quadruple mutant exhibited increased H3K27me3 signals compared with WT at the *FLC* locus (Fig. 3a), consistent with reduced *FLC* expression and accelerated flowering of *dek* (Fig. 2b–d). We examined the whole‐genome distribution of H3K27me3 signals toward histone H3 signals and identified 3998 peaks for WT, 4434 for *dek*, 1085 for *lhp1*, 2050 for *lhp1;dek* (Fig. 3b). The *dek* mutant exhibited a shift in H3K27me3 distribution toward higher signal intensities than the WT (Fig. 2b; Kruskal–Wallis rank sum test; *P*‐value < 2.2e‐16). When we compared the H3K27me3 intensity around CLF‐ or SWN‐binding sites, *dek* showed the highest signal among the examined genotypes (Fig. 3c). As expected, H3K27me3 was reduced in *lhp1*, while the *lhp1;dek* mutant had intermediate levels of H3K27me3 between WT and *lhp1* (Fig. 3b). Furthermore, we visualized the overlap of the gene subset with H3K27me3 in each genotype (Fig. 3d). The largest intersection (2801 genes) is shared between WT and *dek*. The next largest intersection was the *dek*‐unique gene subset. The gene list is provided in Dataset S1. Thus, the deficiency of DEK caused increased H3K27me3, and suppressed the H3K27me3 reduction caused by the loss of *lhp1* in *lhp1;dek*.

To further evaluate the differences in H3K27me3 distribution among the tested genotypes, we plotted the difference of H3K27me3 signal intensity between WT and the different mutants (Fig. 3e–h). The difference of H3K27me3 in *dek* showed an inverse correlation with those in *lhp1*, revealing that genes that lost H3K27me3 in *lhp1* gained H3K27me3 in *dek* (Fig. 3e). Between *lhp1* and *lhp1;dek*, there was a strongly positive correlation of H3K27me3 differences, consistent with the observation that both mutants had lower H3K27me3 levels than WT (Fig. 3f). However, we observed a modest negative correlation when plotting differences between *lhp1;dek* and *lhp1* compared with differences between *lhp1* and WT, consistent with the observation that loci gained H3K27me3 in *lhp1;dek* compared with *lhp1* (Fig. 3g). Between *dek* and *lhp1;dek*, the trend of the correlation was not only less pronounced but also negative (Fig. 3h). The subset of genes that showed alterations of H3K27me3 in *lhp1* was similar to those in *dek*, but the changes occurred in the opposite direction. Thus, PRC2‐target genes gained H3K27me3 in *dek* and the *lhp1;dek* partially neutralizing the loss of H3K27me3 in *lhp1*.

### Invasion of H3K27me3 into pericentromeric telobox‐dense regions in *dek*

**Fig. 4:**
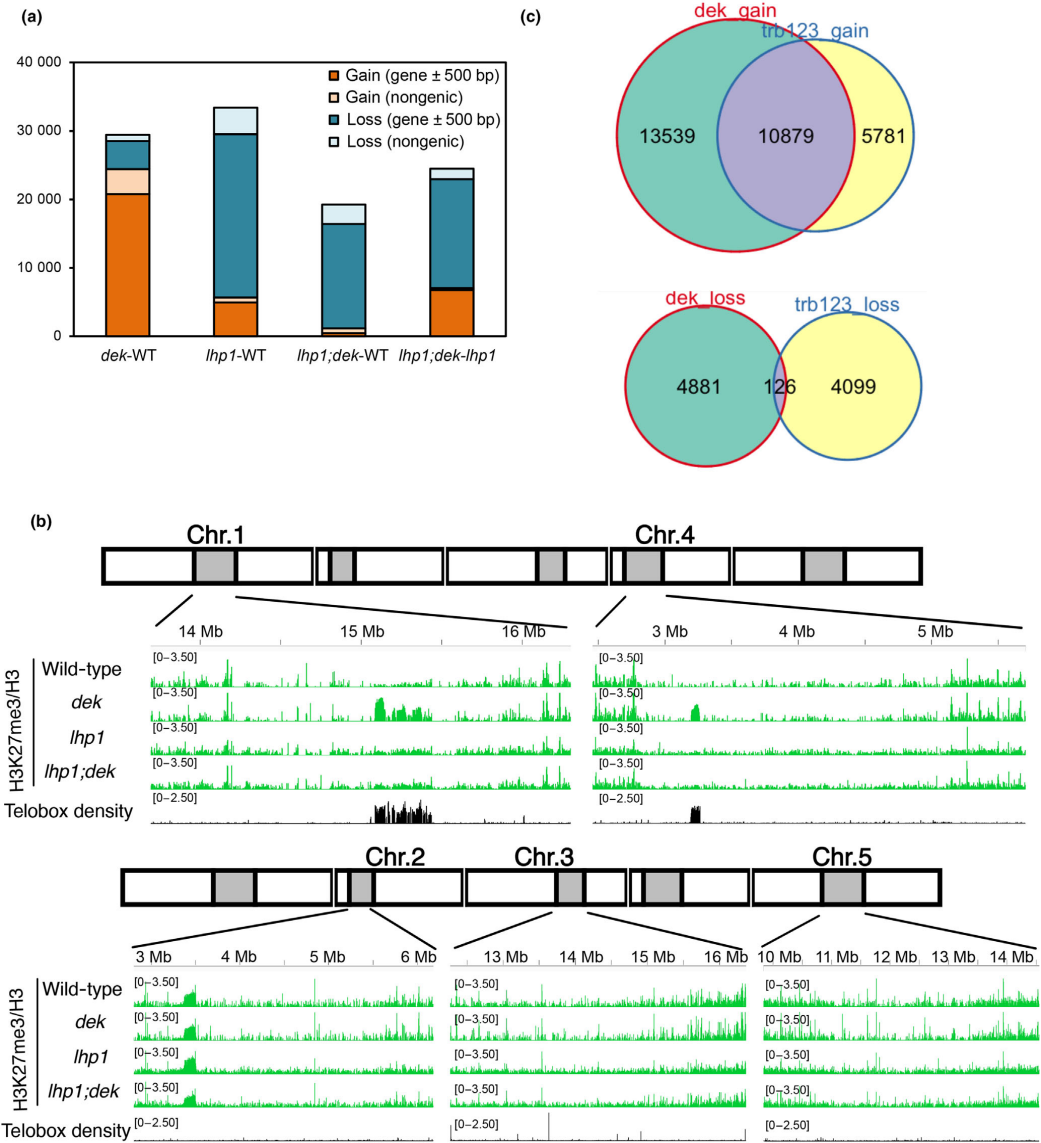
*dek* mutant gains H3K27me3 at telobox‐dense pericentromeric interstitial telomeric repeat regions. (a) Counts and ratios of H3K27me3 gain and loss per 1‐kb bin (sliding window of 500 bp) between two genotypes. Normalized coverage differences > 0.5 and < −0.5 were considered gains and losses, respectively. Due to the 500‐bp sliding window approach, some regions may be counted more than once. (b) Chromosome 1 and 4 have telobox‐dense regions at their pericentromere gaining H3K27me3 in *dek*. Chromosome 2, 3, and 5 do not have telobox‐dense regions nor do they gain H3K27me3 in pericentromeric regions in *dek*. (c) Venn diagram showing overlap of 1‐kb bins with H3K27me3 alterations between *dek* and *trb123*.

We addressed the question of whether the observed increase in H3K27me3 was restricted to PRC2 target genes or also occurred at other regions of the genome. To quantify genome‐wide changes in H3K27me3, we identified 1‐kb bins with 500‐bp sliding windows that gained or lost H3K27me3 (Fig. 4a). In the *dek* mutant, bins with increased H3K27me3 markedly outnumbered those that lost this modification. While most H3K27me3 changes were limited to genic regions (Fig. 4a), we found two pericentromeric regions on Chromosome 1 and 4 that exhibited ectopic H3K27me3 peaks in the *dek* mutant (Figs 4b, S5a,b). In contrast to genic regions, these regions had no H3K27me3 signals in the WT background. These peaks were mitigated in *lhp1;dek*. To identify which sequences were associated with the pericentromeric gain of H3K27me3, we used repeat sequence analysis using the TRASH software (Wlodzimierz *et al.,* 2023). These regions were found to contain highly frequent 7‐ to 8‐bp repeats that correspond to telobox motifs, which are recognized by telomere‐repeat‐binding (TRBs) factors. Specifically, Chromosome 1 and 4 are known to contain large pericentromeric ITR domains (Teano *et al*., 2023), consistent with our observation of increased H3K27me3 at pericentromeric regions in both chromosomes. Interestingly, a similar observation has been made in the histone H1 depleted *h1.1 h1.2* double mutant (referred to as *h1* mutant) (Teano *et al*., 2023), suggesting that loss of DEK, similar to the loss of linker histone H1, allows access for PRC2 to regions that are generally kept inaccessible. We also tested whether *dek* and *h1* mutants have similar changes in H3K27me3 in genic regions. Using publicly available data that were generated from the *h1* mutant, we analyzed which regions gained and lost H3K27me3 (Fig. S6a). As previously reported, we found a comparable number of loci that gained and lost H3K27me3, differing from the preferential gain of H3K27me3 observed in the *dek* mutant (Fig. 4a). Furthermore, there was no significant correlation of H3K27me3 changes in genic regions between both genotypes (Fig. S6b), suggesting distinct connections of H1 and DEK to PRC2 function.

The gain of H3K27me3 in ITR domains in the *h1* mutant has been connected to permissive TRB targeting and recruitment of PRC2 to these regions (Teano *et al*., 2023; Wang *et al*., 2023). We thus speculated that DEK proteins antagonize aberrant TRB‐mediated PRC2 recruitment. We tested this hypothesis by comparing regions that gained or lost H3K27me3 in *dek* and *trb123* using the published *trb123* mutant dataset (Wang *et al*., 2023). Our analysis revealed a substantial overlap between genes gaining H3K27me3 in *dek* mutants with those in *trb123* mutants: *c*. 68% of genes that gained H3K27me3 in *trb123* and 45% of genes that gain H3K27me3 in *dek* reciprocally overlapped (hypergeometric test; *P* < 2.2e‐16) (Fig. 4c). These data reveal that DEK and TRBs independently intersect in regulating PRC2‐mediated chromatin states and that ectopic H3K27me3 in *dek* is not a consequence of aberrant TRB recruitment.

### The synergistic effect between *lhp1* and *dek* is manifested by H3K27me3 target gene deregulation

To further investigate the function of DEKs in gene regulation and to reveal the underlying factors contributing to the enhanced *lhp1;dek* morphological phenotype, we generated strand‐specific RNA transcriptome data of *dek*, *lhp1;dek*, and *lhp1* seedlings. Since ATRX physically interacts with LHP1 (Wang *et al*., 2018), we included transcriptome data from *atrx* mutants to investigate potential functional relationships between ATRX, DEK, and LHP1 in chromatin regulation. ATRX and DEK may have opposing effects on chromatin organization: ATRX deposits H3.3, which antagonizes H1 incorporation (Wollmann *et al*., 2017). We therefore compared the transcriptomes of *atrx*, *dek*, and *lhp1* mutants to examine whether these chromatin differences result in distinct transcriptional outcomes.

**Fig. 5:**
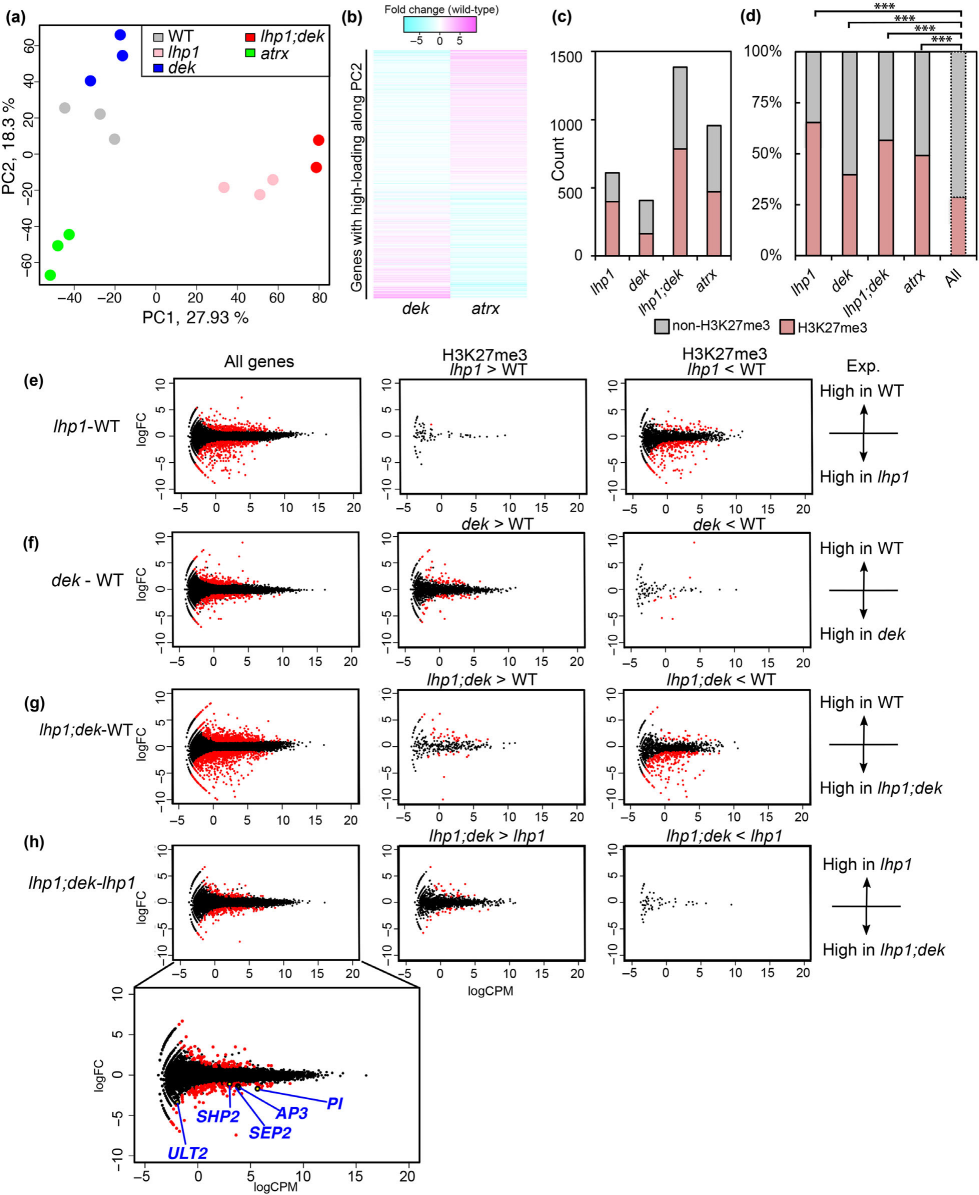
H3K27me3 changes significantly affect gene expression. (a) Plot of Components 1 and 2 of principal component analysis (PCA) based on read counts per gene. Dots having the same color are biological replicates of the same genotypes. (b) Heatmap of gene expression in *dek* and *atrx* with high absolute loading scores on PC2. (c, d) Number of differentially expressed genes (DEGs) and number of H3K27me3 target genes among them in each mutant (c). A 100% stacked bar chart (d). *** indicates *P*‐values < 0.0001 in hypergeometric test. (e–h) MA plots of gene expression grouped by differential H3K27me3 signal between two different genotypes: *lhp1* vs wild‐type (WT) (e), *dek* vs WT (f), *lhp1;dek* vs WT (g), and *lhp1;dek* vs *lhp1* (h). *X*‐axis (log counts per million: logCPM) indicates average expression level on a log scale across two genotypes. *Y*‐axis (log fold change: logFC) indicates fold change between two genotypes on a log scale. DEGs are shown in red color.

PCA was performed to assess sample clustering and identify major sources of variation in gene expression. Biological replicates from the same genotypes clustered well together (Fig. 5a). The first PC (PC1) discriminated *lhp1* and *lhp1;dek* from WT, with *lhp1;dek* being most distant from WT, corresponding to the most severe phenotype of *lhp1;dek*. To assess the biological features associated with each PC, we performed GO enrichment analysis on genes with high absolute loading scores. These loading scores, which indicate how strongly the expression of each gene contributes to a given PC regardless of direction (positive or negative), were considered high when they exceeded three times the SD for that PC. This threshold ensured that the gene subsets had a high contribution to the variance of each PC. The enriched GO terms in PC1 were related to ‘developmental processes’ and ‘response‐to‐stimulus’ (Table S2). PC1 did not discriminate *dek* mutants from WT, consistent with the WT‐like phenotype of *dek* mutants. By contrast, PC2, which represents the second‐largest variance, discriminated *dek* quadruple mutants from the other genotypes. Along the PC2, *atrx* was located on the opposite side compared with *dek* (Fig. 5b), consistent with the idea that loss of ATRX may have opposing consequences to loss of DEK function.

Next, we identified DEGs between mutants and WT based on an absolute log2 fold change > 1 and a FDR of < 0.01. We identified a total of 408, 1387, and 612 DEGs in the *dek* quadruple, the *lhp1;dek*, and the *lhp1* mutants, respectively. In addition, we found a total of 956 DEGs in the *atrx* mutant. Corresponding to the strong morphological phenotype of *lhp1;dek*, the number of DEGs in the *lhp1;dek* mutant was the highest among the examined mutants, resulting in more than twice the number of genes that were differentially expressed compared with *lhp1* (Fig. 5c). We did not observe any drastic changes in antisense transcripts. Therefore, we focused on sense transcripts in subsequent analyses. Nearly half (39.7–65.2%) of the DEGs in all genotypes were H3K27me3 marked genes in WT, which was significantly more than expected by chance (hypergeometric test; *P*‐values = 6.58e‐81 for DEGs in *lhp1*, 6.82e‐07 for DEGs in *dek*, 4.10e‐111 for DEGs in *lhp1;dek*, 8.073e‐43 for DEGs in *atrx*; Fig. 5d). This reveals that in these mutants, genes marked by H3K27me3 were preferentially affected. The larger number of DEGs in *lhp1;dek* was consistent with the enhanced phenotype (Fig. 2), but inconsistent with the partially restored H3K27me3 level compared with the *lhp1* mutant. To explore the connection between gene expression and H3K27me3, we plotted the changes in expression level between each genotype for each gene and then evaluated the H3K27me3 effect in relation to transcript changes. We further refined this analysis by separating plots for genes with increased and decreased H3K27me3 levels between two genotypes. Genes with reduced H3K27me3 in *lhp1* overlapped well with upregulated genes in *lhp1* (205 genes, hypergeometric test; adjusted *P*‐values = 2.71e‐74) (Fig. 5e). Conversely, genes gaining H3K27me3 in *dek* overlapped with downregulated genes in *dek* (57 genes, hypergeometric test; *P*‐values =1.03e‐11) (Fig. 5f). In *lhp1;dek*, there was a significant overlap between genes with decreased H3K27me3 and increased expression (244 genes, hypergeometric test; *P*‐values = 4.19e‐69) (Fig. 5g).

To investigate why the *dek* mutation strongly enhanced the abnormal phenotypes in the *lhp1* background, we identified DEGs in *lhp1;dek* compared with *lhp1*. A total of 239 DEGs were identified, including 151 upregulated and 88 downregulated genes. Among these, 48 DEGs overlapped with genes showing reduced H3K27me3 levels in *lhp1;dek*. Of these, 24 were upregulated and 24 were downregulated (Fig. 5h). Notably, no DEGs overlapped with regions showing increased H3K27me3 in *lhp1;dek* compared with *lhp1*. These findings reveal that DEGs were distributed equally between up‐ and downregulated genes, indicating that, despite the overall increase in H3K27me3, gene expression was not restored to normal levels in the *lhp1;dek* mutant. Interestingly, we found the trithorax group gene *ULTRAPETALA2* (*ULT2*) among those upregulated genes in *lhp1;dek* compared with *lhp1* (Fig. 5h). Overexpression of *ULT2* results in misregulation of MADS‐box genes, which are targeted by the PRC2 (Monfared *et al*., 2013). Consistently, we observed increased expression of MADS‐box genes *SEP2*, *SHP2*, *AP3*, and *PI* in *lhp1;dek* compared with *lhp1*. Taken together, H3K27me3 target genes were cumulatively affected in *lhp1;dek*, regardless of the restoring effect of the *dek* mutation on the genome‐wide H3K27me3 level.

## Discussion

### Impact of DEKs on genome‐wide H3K27me3

Our data revealed that loss of DEK function results in the genome‐wide increase of H3K27me3 in Arabidopsis, suggesting that DEK proteins regulate H3K27me3 deposition or maintenance. Thus, our study brings new insights into the role of DEKs on chromatin function *in vivo*.

Consistent with the interaction between DEK and LHP1 proteins, our data reveal that these genes also genetically interact. We observed a strong enhancement of morphological abnormalities in the quadruple mutant *lhp1;dek*, although this mutant partially suppressed the H3K27me3 loss of the *lhp1* single mutant. While these two observations may not seem to align, transcriptome analysis revealed that the enhancement of developmental defects aligned with the overexpression of the trithorax group gene *ULT2*, which generally leads to gene activation and loss of H3K27me3 (Monfared *et al*., 2013). This overexpression might result in cumulative perturbation of H3K27me3 target gene expression in *lhp1;dek*. Hence, the enhanced morphological abnormalities in *lhp1;dek* possibly involve a multilayered mechanism acting on PRC2 target genes. Alternatively, given that DEK and LHP1 are both implicated in the topological organization of chromatin and/or nucleosomes, their simultaneous loss may compromise higher order chromatin structure, leading to deregulation of gene expression.

While the *dek* mutants showed little morphological aberrations under normal conditions, previous work reported that the *dek3* mutant exhibits salt‐stress hypersensitivity (Waidmann *et al*., 2014). Additionally, overexpression of *DEK3* causes increased sensitivity to cold stress (Brestovitsky *et al*., 2019). Interestingly, the increase in genome‐wide H3K27me3 has also been reported in WT plants under environmental stress conditions, such as warm temperature (Yamaguchi *et al*., 2021; Kim *et al*., 2023). Considering these recent reports showing stress‐induced H3K27me3 changes, DEK proteins may act to maintain appropriate H3K27me3 levels under fluctuating environmental conditions. Previous *in vitro* studies of human DEK proteins revealed that they affect DNA topology (Waldmann *et al*., 2002, 2004; Kujirai *et al*., 2025). However, the molecular functions *in vivo* underlying these observations had been unclear. Our findings point to the possibility that this topology regulation by DEK might be important under environmental stress through chromatin regulation.

### Similarity and difference between *dek* and *h1* mutants

In our study, defects in *dek* caused increased H3K27me3 in both genic regions and ITRs at the pericentromere of Chromosome 1 and 4. While this observation looks contradictory to the previous study from Kujirai *et al*. (2025), who reported that DEK facilitates H3K27me3 deposition *in vitro*, the discrepancy likely reflects differences in experimental systems. Our observations are based on *in vivo* studies in Arabidopsis, whereas their findings are based on a reconstituted *in vitro* system. Given that *in vivo* there are several different layers of H3K27me3 regulation, including LHP1, trithorax group proteins, and histone variants, the increase of H3K27me3 in *dek* may result from the combined outcomes of several different factors.

Intriguingly, our finding that H3K27me3 accumulated at pericentromeric ITRs in *dek* resembles observations made in histone *h1* mutants (Teano *et al*., 2023). However, unlike in the *dek* mutant, the *h1* mutant exhibited loss of H3K27me3 at many genic regions. Thus, while *dek* and *h1* mutations both result in H3K27me3 gain at ITRs, their effects on genic regions are opposite (Fig. 3) (Teano *et al*., 2023). Notably, ectopic gain of H3K27me3 at ITRs was absent in *lhp1;dek*, suggesting that gain of H3K27me3 in *dek* depends on functional LHP1. This finding may also reconcile the contradictory observation of DEK's effect on H3K27me3 between *in vivo* and *in vitro*. Although both H1 and DEK are involved in chromatin compaction (Teano *et al*., 2023; Kujirai *et al*., 2025), Kujirai *et al*. found that human H1 and DEK restrict linker DNA into distinct orientations. Therefore, the chromatin compaction mediated by histone H1 and DEK may lead to a structurally different chromatin status, causing different outcomes on H3K27me3 levels in genic regions. In genic regions, H3K27me3 increased specifically at sites already marked in WT, consistent with DEK colocalizing with H3K27me3 (Kujirai *et al*., 2025). This suggests DEK maintains proper H3K27me3 levels rather than simply promoting deposition. We propose that DEK modulates LHP1 function through its effects on linker DNA topology: At *bona fide* Polycomb targets, DEK and LHP1 cooperate to maintain stable H3K27me3 domains, whereas at ITRs, DEK prevents inappropriate LHP1 and/or PRC2 activity. Loss of DEK disrupts this balance, allowing ectopic compaction at normally protected regions.

### Potential roles of DEK in gene expression and chromatin regulation contrasting to ATRX

In animals, loss of DEK causes an imbalance between histone variant H3.3 and H3.1 (Ivanauskiene *et al*., 2014). Our previous genome‐wide analysis showed that H3.3 signals are associated with genes that also carry H3K27me3 (Shu *et al*., 2014). It is tempting to speculate that increased H3K27me3 in *dek* is caused by the abnormal deposition of H3.3. For instance, ATRX is a protein that potentially facilitates H3.3 incorporation. It has been reported that loss of ATRX in Arabidopsis causes *FLC* overexpression and Arabidopsis ATRX is associated with LHP1 (Wang *et al*., 2018). While *FLC* is upregulated in the *atrx* mutant, it is downregulated in *dek* mutants (Wang *et al*., 2018; Zong *et al*., 2021) (Fig. 2d). Thus, defects in DEK and ATRX show inverse effects on *FLC* expression. Furthermore, the transcriptome profiles of these two mutants showed that many other genes were also affected in the opposite direction in *atrx* and *dek* (Fig. 5c,d). Thus, DEK and ATRX both interact with LHP1, and their deficiencies cause opposing effects on their transcriptome, implying cross‐talk between DEK and ATRX. We speculate that the connection could be histone H1, which is negatively affected upon H3.3 incorporation and is expected to accumulate ectopically in *atrx*. By contrast, loss of DEK function may impair chromatin compaction mediated by histone H1. Further studies are required to elucidate the molecular mechanism underlying these opposing effects. Taken together, our findings highlight DEK as a novel factor affecting genome‐wide histone H3K27me3 levels *in vivo*.

In conclusion, our study revealed the importance of DEK in plant development and histone H3K27me3 modification. This work not only clarifies the *in vivo* functions of DEK but also provides insights into the molecular mechanism for the multilayered regulation of H3K27me3.

## Competing interests

None declared.

## Author contributions

MN, MD, LH, KL and CK conceived and designed the experiments. MN, HB, MD, MG, KL and MT performed the experiments. MN, HB, MD, KL and MT analyzed the data. ES and CK were involved in technical advice and discussion. MN, CK, MT and KL wrote the paper.

## Disclaimer

The New Phytologist Foundation remains neutral with regard to jurisdictional claims in maps and in any institutional affiliations.

## Supporting information


**Dataset S1** Gene lists for H3K27me3 peak overlaps between genotypes.

**Fig. S1** Molecular verification of *ppdekl* knockout lines.
**Fig. S2** DEK proteins are conserved between animals and plants.
**Fig. S3** *dek* T‐DNA mutants examined in this study.
**Fig. S4** *DEKs* and *LHP1* coexpress and their proteins interact in nuclei.
**Fig. S5** Extended and detailed views of H3K27me3 distribution at interstitial telomeric repeat (ITR) genomic region.
**Fig. S6** Comparison of H3K27me3 changes in the *dek* and the *h1* mutants.
**Table S1** Primer sequences for genotyping RT‐qPCR, ChIP, and semi‐quantitative for DEK transcripts and plasmid construction.
**Table S2** Gene Ontology enrichment and adjusted *P*‐values of genes with high loadings on the Principal component 1 (PC1).
Please note: Wiley is not responsible for the content or functionality of any Supporting Information supplied by the authors. Any queries (other than missing material) should be directed to the *New Phytologist* Central Office.

## Data Availability

Raw and processed sequencing data of RNA‐seq and ChIP‐seq are available in the NCBI Gene Expression Omnibus (https://www.ncbi.nlm.nih.gov/geo/) as accession no. GSE175373.

## References

[nph70704-bib-0001] Abba MC , Sun H , Hawkins KA , Drake JA , Hu Y , Nunez MI , Gaddis S , Shi T , Horvath S , Sahin A *et al*. 2007. Breast cancer molecular signatures as determined by SAGE: correlation with lymph node status. Molecular Cancer Research 5: 881–890. 17855657 10.1158/1541-7786.MCR-07-0055 PMC4186709

[nph70704-bib-0002] Alexiadis V , Waldmann T , Andersen J , Mann M , Knippers R , Gruss C . 2000. The protein encoded by the proto‐oncogene DEK changes the topology of chromatin and reduces the efficiency of DNA replication in a chromatin‐specific manner. Genes & Development 14: 1308–1312. 10837023 PMC316669

[nph70704-bib-0003] Anders S , Pyl PT , Huber W . 2015. HTSeq–a Python framework to work with high‐throughput sequencing data. Bioinformatics 31: 166–169. 25260700 10.1093/bioinformatics/btu638 PMC4287950

[nph70704-bib-0004] Böhm F , Kappes F , Scholten I , Richter N , Matsuo H , Knippers R , Waldmann T . 2005. The SAF‐box domain of chromatin protein DEK. Nucleic Acids Research 33: 1101–1110. 15722484 10.1093/nar/gki258 PMC549417

[nph70704-bib-0005] Brestovitsky A , Ezer D , Waidmann S , Maslen SL , Balcerowicz M , Cortijo S , Charoensawan V , Martinho C , Rhodes D , Jonak C *et al*. 2019. DEK influences the trade‐off between growth and arrest via H2A.Z‐nucleosomes in Arabidopsis (Plant Biology). *bioRxiv* . doi: 10.1101/829226.

[nph70704-bib-0006] Carro MS , Spiga FM , Quarto M , Ninni VD , Volorio S , Alcalay M , Müller H . 2006. DEK expression is controlled by E2F and deregulated in diverse tumor type. Cell Cycle 5: 1202–1207. 16721057 10.4161/cc.5.11.2801

[nph70704-bib-0007] Derkacheva M , Liu S , Figueiredo DD , Gentry M , Mozgova I , Nanni P , Tang M , Mannervik M , Köhler C , Hennig L . 2016. H2A deubiquitinases UBP12/13 are part of the Arabidopsis polycomb group protein system. Nature Plants 2: 16126. 27525512 10.1038/nplants.2016.126

[nph70704-bib-0008] Drané P , Ouararhni K , Depaux A , Shuaib M , Hamiche A . 2010. The death‐associated protein DAXX is a novel histone chaperone involved in the replication‐independent deposition of H3.3. Genes & Development 24: 1253–1265. 20504901 10.1101/gad.566910 PMC2885661

[nph70704-bib-0009] Exner V , Aichinger E , Shu H , Wildhaber T , Alfarano P , Caflisch A , Gruissem W , Köhler C , Hennig L . 2009. The chromodomain of LIKE HETEROCHROMATIN PROTEIN 1 is essential for H3K27me3 binding and function during Arabidopsis development. PLoS ONE 4: e5335. 19399177 10.1371/journal.pone.0005335 PMC2670505

[nph70704-bib-0010] Gaudin V , Libault M , Pouteau S , Juul T , Zhao G , Lefebvre D , Grandjean O . 2001. Mutations in LIKE HETEROCHROMATIN PROTEIN 1 affect flowering time and plant architecture in Arabidopsis. Development (Cambridge, England) 128: 4847–4858. 11731464 10.1242/dev.128.23.4847

[nph70704-bib-0011] Goldberg AD , Banaszynski LA , Noh K‐M , Lewis PW , Elsaesser SJ , Stadler S , Dewell S , Law M , Guo X , Li X *et al*. 2010. Distinct factors control histone variant H3.3 localization at specific genomic regions. Cell 140: 678–691. 20211137 10.1016/j.cell.2010.01.003 PMC2885838

[nph70704-bib-0013] Grasemann C , Gratias S , Stephan H , Schüler A , Schramm A , Klein‐Hitpass L , Rieder H , Schneider S , Kappes F , Eggert A *et al*. 2005. Gains and overexpression identify DEK and E2F3 as targets of chromosome 6p gains in retinoblastoma. Oncogene 24: 6441–6449. 16007192 10.1038/sj.onc.1208792

[nph70704-bib-0014] Grefen C , Donald N , Hashimoto K , Kudla J , Schumacher K , Blatt MR . 2010. A ubiquitin‐10 promoter‐based vector set for fluorescent protein tagging facilitates temporal stability and native protein distribution in transient and stable expression studies: fluorescence tagging and expression in Arabidopsis. The Plant Journal 64: 355–365. 20735773 10.1111/j.1365-313X.2010.04322.x

[nph70704-bib-0015] Hiss M , Meyberg R , Westermann J , Haas FB , Schneider L , Schallenberg‐Rüdinger M , Ullrich KK , Rensing SA . 2017. Sexual reproduction, sporophyte development and molecular variation in the model moss *Physcomitrella patens*: introducing the ecotype Reute. The Plant Journal 90: 606–620. 28161906 10.1111/tpj.13501

[nph70704-bib-0016] Hollenbach AD , McPherson CJ , Mientjes EJ , Iyengar R , Grosveld G . 2002. Daxx and histone deacetylase II associate with chromatin through an interaction with core histones and the chromatin‐associated protein Dek. Journal of Cell Science 115: 3319–3330. 12140263 10.1242/jcs.115.16.3319

[nph70704-bib-0017] Ivanauskiene K , Delbarre E , McGhie JD , Küntziger T , Wong LH , Collas P . 2014. The PML‐associated protein DEK regulates the balance of H3.3 loading on chromatin and is important for telomere integrity. Genome Research 24: 1584–1594. 25049225 10.1101/gr.173831.114 PMC4199371

[nph70704-bib-0018] Kappes F , Waldmann T , Mathew V , Yu J , Zhang L , Khodadoust MS , Chinnaiyan AM , Luger K , Erhardt S , Schneider R *et al*. 2011. The DEK oncoprotein is a Su(var) that is essential to heterochromatin integrity. Genes & Development 25: 673–678. 21460035 10.1101/gad.2036411 PMC3070930

[nph70704-bib-0019] Kim D , Paggi JM , Park C , Bennett C , Salzberg SL . 2019. Graph‐based genome alignment and genotyping with HISAT2 and HISAT‐genotype. Nature Biotechnology 37: 907–915. 10.1038/s41587-019-0201-4 PMC7605509 31375807

[nph70704-bib-0020] Kim J , Bordiya Y , Xi Y , Zhao B , Kim D‐H , Pyo Y , Zong W , Ricci WA , Sung S . 2023. Warm temperature‐triggered developmental reprogramming requires VIL1‐mediated, genome‐wide H3K27me3 accumulation in *Arabidopsis* . Development 150: dev201343. 36762655 10.1242/dev.201343 PMC10110417

[nph70704-bib-0021] Kipp M , Göhring F , Ostendorp T , van Drunen CM , van Driel R , Przybylski M , Fackelmayer FO . 2000. SAF‐box, a conserved protein domain that specifically recognizes scaffold attachment region DNA. Molecular and Cellular Biology 20: 7480–7489. 11003645 10.1128/mcb.20.20.7480-7489.2000 PMC86301

[nph70704-bib-0022] Kolberg L , Raudvere U , Kuzmin I , Adler P , Vilo J , Peterson H . 2023. g:Profiler—interoperable web service for functional enrichment analysis and gene identifier mapping (2023 update). Nucleic Acids Research 51(W1): W207–W212. 37144459 10.1093/nar/gkad347 PMC10320099

[nph70704-bib-0023] Kujirai T , Echigoya K , Kishi Y , Saeki M , Ito T , Kato J , Negishi L , Kimura H , Masumoto H , Takizawa Y *et al*. 2025. Structural insights into how DEK nucleosome binding facilitates H3K27 trimethylation in chromatin. Nature Structural & Molecular Biology 32: 1183–1192. 10.1038/s41594-025-01493-w PMC12263440 39984731

[nph70704-bib-0024] Kumar S , Stecher G , Tamura K . 2016. Mega7: molecular evolutionary genetics analysis version 7.0 for bigger datasets. Molecular Biology and Evolution 33: 1870–1874. 27004904 10.1093/molbev/msw054 PMC8210823

[nph70704-bib-0025] Larramendy ML , Niini T , Elonen E , Nagy B , Ollila J , Vihinen M , Knuutila S . 2002. Overexpression of translocation‐associated fusion genes of FGFRI, MYC, NPMI, and DEK, but absence of the translocations in acute myeloid leukemia. A microarray analysis. Haematologica 87: 569–577. 12031912

[nph70704-bib-0026] Lewis PW , Elsaesser SJ , Noh K‐M , Stadler SC , Allis CD . 2010. Daxx is an H3.3‐specific histone chaperone and cooperates with ATRX in replication‐independent chromatin assembly at telomeres. Proceedings of the National Academy of Sciences, USA 107: 14075–14080. 10.1073/pnas.1008850107 PMC2922592 20651253

[nph70704-bib-0027] Li Q , Brown JB , Huang H , Bickel PJ . 2011. Measuring reproducibility of high‐throughput experiments. The Annals of Applied Statistics 5: 1752–1779.

[nph70704-bib-0028] Lid SE , Gruis D , Jung R , Lorentzen JA , Ananiev E , Chamberlin M , Niu X , Meeley R , Nichols S , Olsen O‐A . 2002. The defective kernel 1 (dek1) gene required for aleurone cell development in the endosperm of maize grains encodes a membrane protein of the calpain gene superfamily. National Academy of Sciences of the United States of America 99: 5460–5465. 10.1073/pnas.042098799 PMC122791 11929961

[nph70704-bib-0029] von Lindern M , Fornerod M , van Baal S , Jaegle M , de Wit T , Buijs A , Grosveld G . 1992. The translocation (6;9), associated with a specific subtype of acute myeloid leukemia, results in the fusion of two genes, dek and can, and the expression of a chimeric, leukemia‐specific dek‐can mRNA. Molecular and Cellular Biology 12: 1687–1697. 1549122 10.1128/mcb.12.4.1687-1697.1992 PMC369612

[nph70704-bib-0030] Luo C , Lam E . 2014. Quantitatively profiling genome‐wide patterns of histone modifications in *Arabidopsis thaliana* using ChIP‐seq. In: Spillane C , McKeown PC , eds. Plant epigenetics and epigenomics. Methods in molecular biology. Totowa, NJ: Humana Press, 177–193. doi: 10.1007/978-1-62703-773-0_12. 24478015

[nph70704-bib-0031] Martin K , Kopperud K , Chakrabarty R , Banerjee R , Brooks R , Goodin MM . 2009. Transient expression in *Nicotiana benthamiana* fluorescent marker lines provides enhanced definition of protein localization, movement and interactions *in planta* . The Plant Journal 59: 150–162. 19309457 10.1111/j.1365-313X.2009.03850.x

[nph70704-bib-0032] Martin M . 2011. Cutadapt removes adapter sequences from high‐throughput sequencing reads. EMBnet Journal 17: 10–12.

[nph70704-bib-0033] Monfared MM , Carles CC , Rossignol P , Pires HR , Fletcher JC . 2013. The ULT1 and ULT2 trxG genes play overlapping roles in Arabidopsis development and gene regulation. Molecular Plant 6: 1564–1579. 23446032 10.1093/mp/sst041

[nph70704-bib-0034] Murashige T , Skoog F . 1962. A revised medium for rapid growth and bio assays with tobacco tissue cultures. Physiologia Plantarum 15: 473–497.

[nph70704-bib-0035] Notredame C , Higgins DG , Heringa J . 2000. T‐coffee: a novel method for fast and accurate multiple sequence alignment 1 1Edited by J. Thornton. Journal of Molecular Biology 302: 205–217. 10964570 10.1006/jmbi.2000.4042

[nph70704-bib-0036] Parihar V , Arya D , Walia A , Tyagi V , Dangwal M , Verma V , Khurana R , Boora N , Kapoor S , Kapoor M . 2019. Functional characterization of LIKE HETEROCHROMATIN PROTEIN 1 in the moss *Physcomitrella patens*: its conserved protein interactions in land plants. The Plant Journal 97: 221–239. 30537172 10.1111/tpj.14182

[nph70704-bib-0037] Pendle AF , Clark GP , Boon R , Lewandowska D , Lam YW , Andersen J , Mann M , Lamond AI , Brown JWS , Shaw PJ . 2005. Proteomic analysis of the *Arabidopsis* nucleolus suggests novel nucleolar functions. Molecular Biology of the Cell 16: 260–269. 15496452 10.1091/mbc.E04-09-0791 PMC539170

[nph70704-bib-0038] Perroud P , Demko V , Johansen W , Wilson RC , Olsen O , Quatrano RS . 2014. Defective Kernel 1 (DEK 1) is required for three‐dimensional growth in *Physcomitrella patens* . New Phytologist 203: 794–804. 24844771 10.1111/nph.12844 PMC4285852

[nph70704-bib-0039] Privette Vinnedge LM , Kappes F , Nassar N , Wells SI . 2013. Stacking the DEK: from chromatin topology to cancer stem cells. Cell Cycle 12: 51–66. 23255114 10.4161/cc.23121 PMC3570517

[nph70704-bib-0040] Quinlan AR , Hall IM . 2010. BEDTools: a flexible suite of utilities for comparing genomic features. Bioinformatics 26: 841–842. 20110278 10.1093/bioinformatics/btq033 PMC2832824

[nph70704-bib-0041] Ramirez‐Prado JS , Latrasse D , Rodriguez‐Granados NY , Huang Y , Manza‐Mianza D , Brik‐Chaouche R , Jaouannet M , Citerne S , Bendahmane A , Hirt H *et al*. 2019. The Polycomb protein lhp 1 regulates *Arabidopsis thaliana* stress responses through the repression of the myc 2‐dependent branch of immunity. The Plant Journal 100: 1118–1131. 31437321 10.1111/tpj.14502

[nph70704-bib-0042] Reimand J , Kull M , Peterson H , Hansen J , Vilo J . 2007. g:Profiler—a web‐based toolset for functional profiling of gene lists from large‐scale experiments. Nucleic Acids Research 35(suppl_2): W193–W200. 17478515 10.1093/nar/gkm226 PMC1933153

[nph70704-bib-0043] Riveiro‐Falkenbach E , Soengas MS . 2010. Control of tumorigenesis and chemoresistance by the DEK oncogene. Clinical Cancer Research 16: 2932–2938. 20501624 10.1158/1078-0432.CCR-09-2330 PMC2931273

[nph70704-bib-0044] Robert X , Gouet P . 2014. Deciphering key features in protein structures with the new ENDscript server. Nucleic Acids Research 42: W320–W324. 24753421 10.1093/nar/gku316 PMC4086106

[nph70704-bib-0045] Robinson JT , Thorvaldsdóttir H , Winckler W , Guttman M , Lander ES , Getz G , Mesirov JP . 2011. Integrative genomics viewer. Nature Biotechnology 29: 24–26. 10.1038/nbt.1754 PMC3346182 21221095

[nph70704-bib-0046] Robinson MD , McCarthy DJ , Smyth GK . 2010. edgeR: a Bioconductor package for differential expression analysis of digital gene expression data. Bioinformatics 26: 139–140. 19910308 10.1093/bioinformatics/btp616 PMC2796818

[nph70704-bib-0047] Saitou N , Nei M . 1987. The neighbor‐joining method: a new method for reconstructing phylogenetic trees. Molecular Biology and Evolution 4: 406–425. 3447015 10.1093/oxfordjournals.molbev.a040454

[nph70704-bib-0048] Sanchez‐Carbayo M , Socci ND , Lozano JJ , Li W , Charytonowicz E , Belbin TJ , Prystowsky MB , Ortiz AR , Childs G , Cordon‐Cardo C . 2003. Gene Discovery in Bladder Cancer Progression using cDNA Microarrays. The American Journal of Pathology 163: 505–516. 12875971 10.1016/S0002-9440(10)63679-6 PMC1868230

[nph70704-bib-0049] Schaefer D , Zryd J‐P , Knight CD , Cove DJ . 1991. Stable transformation of the moss *Physcomitrella patens* . Molecular & General Genetics 226: 418–424. 2038304 10.1007/BF00260654

[nph70704-bib-0050] Schmid M , Davison TS , Henz SR , Pape UJ , Demar M , Vingron M , Schölkopf B , Weigel D , Lohmann JU . 2005. A gene expression map of *Arabidopsis thaliana* development. Nature Genetics 37: 501–506. 15806101 10.1038/ng1543

[nph70704-bib-0051] Shu H , Nakamura M , Siretskiy A , Borghi L , Moraes I , Wildhaber T , Gruissem W , Hennig L . 2014. Arabidopsisreplacement histone variant H3.3 occupies promoters of regulated genes. Genome Biology 15: R62. 24708891 10.1186/gb-2014-15-4-r62 PMC4054674

[nph70704-bib-0052] Shu J , Chen C , Thapa RK , Bian S , Nguyen V , Yu K , Yuan Z‐C , Liu J , Kohalmi SE , Li C *et al*. 2019. Genome‐wide occupancy of histone H3K27 methyltransferases CURLY LEAF and SWINGER in Arabidopsis seedlings. Plant Direct 3: e00100. 31245749 10.1002/pld3.100 PMC6508855

[nph70704-bib-0053] Sundaram R , Gandhi S , Jonak C , Vasudevan D . 2024. Characterization of the *Arabidopsis thaliana* chromatin remodeler DEK3 for its interaction with histones and DNA. Biochimie 227: 248–261. 39097158 10.1016/j.biochi.2024.07.018

[nph70704-bib-0054] Supek F , Bošnjak M , Škunca N , Šmuc T . 2011. REVIGO summarizes and visualizes long lists of gene ontology terms. PLoS ONE 6: e21800. 21789182 10.1371/journal.pone.0021800 PMC3138752

[nph70704-bib-0055] Teano G , Concia L , Wolff L , Carron L , Biocanin I , Adamusová K , Fojtová M , Bourge M , Kramdi A , Colot V *et al*. 2023. Histone H1 protects telomeric repeats from H3K27me3 invasion in Arabidopsis. Cell Reports 42: 112894. 37515769 10.1016/j.celrep.2023.112894

[nph70704-bib-0056] Thelander M , Nilsson A , Olsson T , Johansson M , Girod P‐A , Schaefer DG , Zrÿd J‐P , Ronne H . 2007. The moss genes PpSKI1 and PpSKI2 encode nuclear SnRK1 interacting proteins with homologues in vascular plants. Plant Molecular Biology 64: 559–573. 17533513 10.1007/s11103-007-9176-5

[nph70704-bib-0057] Turck F , Roudier F , Farrona S , Martin‐Magniette M‐L , Guillaume E , Buisine N , Gagnot S , Martienssen RA , Coupland G , Colot V . 2007. Arabidopsis TFL2/LHP1 specifically associates with genes marked by trimethylation of histone H3 lysine 27. PLoS Genetics 3: e86. 17542647 10.1371/journal.pgen.0030086 PMC1885283

[nph70704-bib-0058] Veluchamy A , Jégu T , Ariel F , Latrasse D , Mariappan KG , Kim S‐K , Crespi M , Hirt H , Bergounioux C , Raynaud C *et al*. 2016. LHP1 Regulates H3K27me3 spreading and shapes the three‐dimensional conformation of the Arabidopsis genome. PLoS ONE 11: e0158936. 27410265 10.1371/journal.pone.0158936 PMC4943711

[nph70704-bib-0059] Waidmann S , Kusenda B , Mayerhofer J , Mechtler K , Jonak C . 2014. A DEK domain‐containing protein modulates chromatin structure and function in Arabidopsis. Plant Cell 26: 4328–4344. 25387881 10.1105/tpc.114.129254 PMC4277211

[nph70704-bib-0060] Waldmann T , Baack M , Richter N , Gruss C . 2003. Structure‐specific binding of the proto‐oncogene protein DEK to DNA. Nucleic Acids Research 31: 7003–7010. 14627833 10.1093/nar/gkg864 PMC290247

[nph70704-bib-0061] Waldmann T , Eckerich C , Baack M , Gruss C . 2002. The ubiquitous chromatin protein DEK alters the structure of DNA by introducing positive supercoils. Journal of Biological Chemistry 277: 24988–24994. 11997399 10.1074/jbc.M204045200

[nph70704-bib-0062] Waldmann T , Scholten I , Kappes F , Hu HG , Knippers R . 2004. The DEK protein – an abundant and ubiquitous constituent of mammalian chromatin. Gene 343: 1–9. 15563827 10.1016/j.gene.2004.08.029

[nph70704-bib-0063] Wang H , Jiang D , Axelsson E , Lorković ZJ , Montgomery S , Holec S , Pieters BJGE , Al Temimi AHK , Mecinović J , Berger F . 2018. LHP1 interacts with ATRX through plant‐specific domains at specific loci targeted by PRC2. Molecular Plant 11: 1038–1052. 29793052 10.1016/j.molp.2018.05.004

[nph70704-bib-0064] Wang H , Liu C , Cheng J , Liu J , Zhang L , He C , Shen W‐H , Jin H , Xu L , Zhang Y . 2016. Arabidopsis flower and embryo developmental genes are repressed in seedlings by different combinations of polycomb group proteins in association with distinct sets of *cis*‐regulatory elements. PLoS Genetics 12: e1005771. 26760036 10.1371/journal.pgen.1005771 PMC4711971

[nph70704-bib-0065] Wang M , Zhong Z , Gallego‐Bartolomé J , Feng S , Shih Y‐H , Liu M , Zhou J , Richey JC , Ng C , Jami‐Alahmadi Y *et al*. 2023. Arabidopsis TRB proteins function in H3K4me3 demethylation by recruiting JMJ14. Nature Communications 14: 1736. 10.1038/s41467-023-37263-9 PMC10049986 36977663

[nph70704-bib-0066] Wang Y , Tzfira T , Gaba V , Citovsky V , Palukaitis P , Gal‐On A . 2004. Functional analysis of the cucumber mosaic virus 2b protein: pathogenicity and nuclear localization. Journal of General Virology 85: 3135–3147. 15448377 10.1099/vir.0.80250-0

[nph70704-bib-0067] Wlodzimierz P , Hong M , Henderson IR . 2023. TRASH: tandem repeat annotation and structural hierarchy. Bioinformatics 39: btad308. 37162382 10.1093/bioinformatics/btad308 PMC10199239

[nph70704-bib-0068] Wollmann H , Stroud H , Yelagandula R , Tarutani Y , Jiang D , Jing L , Jamge B , Takeuchi H , Holec S , Nie X *et al*. 2017. The histone H3 variant H3.3 regulates gene body DNA methylation in Arabidopsis thaliana. Genome Biology 18: 94. 28521766 10.1186/s13059-017-1221-3 PMC5437678

[nph70704-bib-0069] Yamaguchi N , Matsubara S , Yoshimizu K , Seki M , Hamada K , Kamitani M , Kurita Y , Nomura Y , Nagashima K , Inagaki S *et al*. 2021. H3K27me3 demethylases alter HSP22 and HSP17.6C expression in response to recurring heat in Arabidopsis. Nature Communications 12: 3480. 10.1038/s41467-021-23766-w PMC8190089 34108473

[nph70704-bib-0070] Zhang X , Clarenz O , Cokus S , Bernatavichute YV , Pellegrini M , Goodrich J , Jacobsen SE . 2007. Whole‐genome analysis of histone H3 lysine 27 trimethylation in Arabidopsis. PLoS Biology 5: e129. 17439305 10.1371/journal.pbio.0050129 PMC1852588

[nph70704-bib-0071] Zhang Y , Liu T , Meyer CA , Eeckhoute J , Johnson DS , Bernstein BE , Nusbaum C , Myers RM , Brown M , Li W *et al*. 2008. Model‐based analysis of ChIP‐Seq (MACS). Genome Biology 9: R137. 18798982 10.1186/gb-2008-9-9-r137 PMC2592715

[nph70704-bib-0072] Zong W , Zhao B , Xi Y , Bordiya Y , Mun H , Cerda NA , Kim D‐H , Sung S . 2021. DEK domain‐containing proteins control flowering time in Arabidopsis. New Phytologist 231: 182–192. 33774831 10.1111/nph.17366 PMC8985477

