# Supplementary material for: Chromatin‐associated DEK proteins maintain H3K27me3 balance and coordinate developmental transitions in plants
Source: New Phytol. 2025 Nov 14;249(2):930–44. doi: 10.1111/nph.70704 (PMC12712427; doi:10.1111/nph.70704)
Supplement: Supplementary file 2 — Fig. S1 Molecular verification of ppdekl knockout lines. Fig. S2 DEK proteins are conserved between animals and plants. Fig. S3 dek T‐DNA mutants examined in this study. Fig. S4 DEKs and LHP1 coexpress and their proteins interact in nuclei. Fig. S5 Extended and detailed views of H3K27me3 distribution at interstitial telomeric repeat (ITR) genomic region. Fig. S6 Comparison of H3K27me3 changes in the dek and the h1 mutants. Table S1 Primer sequences for genotyping RT‐qPCR, ChIP, and semi‐quantitative for DEK transcripts and plasmid construction. Table S2 Gene Ontology enrichment and adjusted P‐values of genes with high loadings on the Principal component 1 (PC1). Please note: Wiley is not responsible for the content or functionality of any Supporting Information supplied by the authors. Any queries (other than missing material) should be directed to the New Phytologist Central Office. [file NPH-249-930-s001.pdf]

***New Phytologist* Supporting Information**

Article title: Chromatin-Associated DEK proteins Maintain H3K27me3 Balance and Coordinate Developmental Transitions in plants

Authors: **Miyuki Nakamura, Heinrich Bente, Maria Derkacheva, Matthew Gentry, Katarina Landberg, Mattias Thelander, Eva Sundberg , Lars Hennig and Claudia Köhler**

Article acceptance date: 13 October 2025

The following Supporting Information is available for this article:

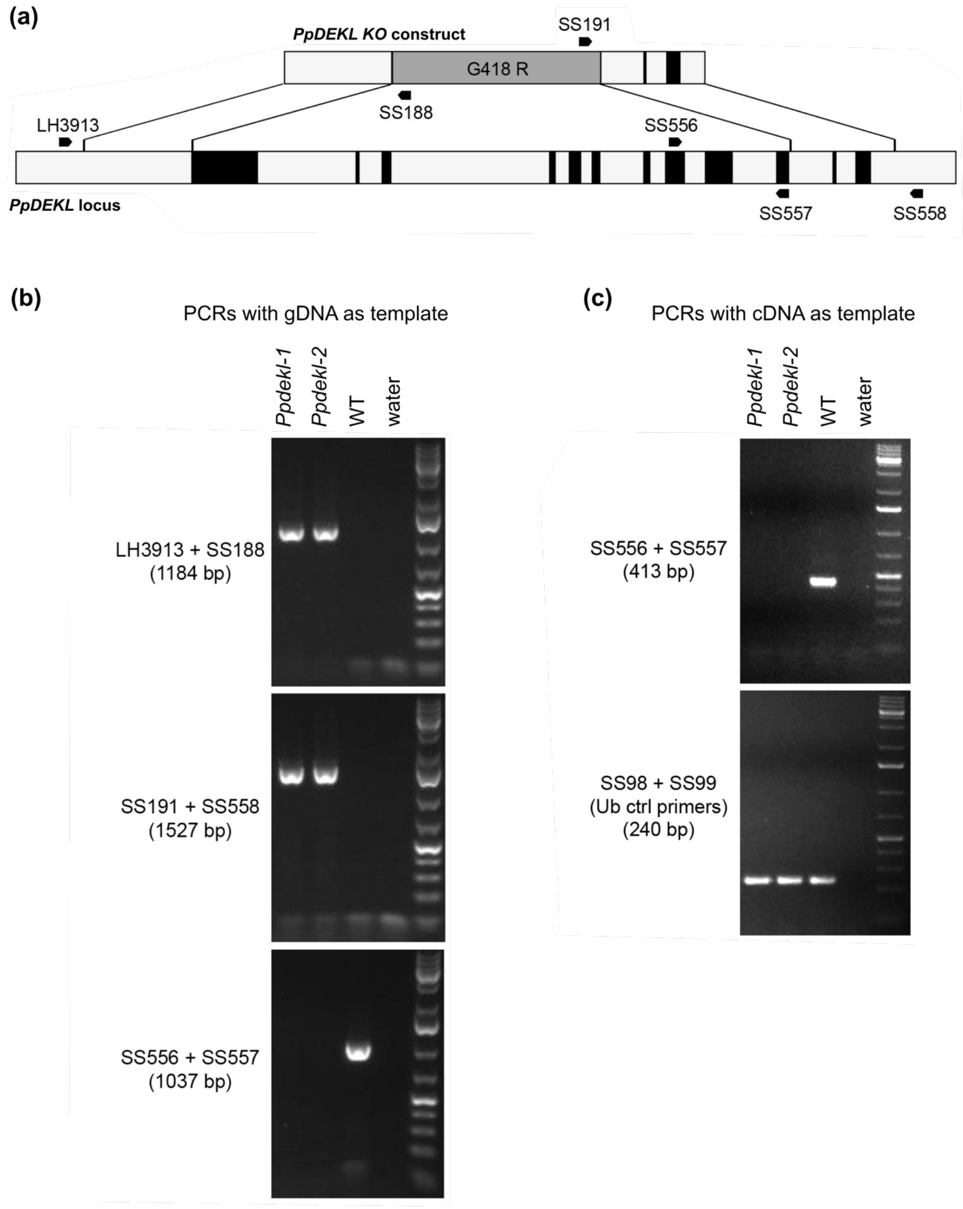

Fig. S1 Molecular verification of *ppdekl* knockout lines

(A) Schematic representation of the wild-type *PpDEKL* locus and the linearized knockout construct used to target it. Coding parts of exons are shown in black, non-coding introns and flanking sequences are shown in white, and the selection cassette in the targeting construct is shown in gray. Approximate annealing sites of primers used for PCR verification in B) and C) are shown as black arrow heads. (B) Results from genotyping PCR's with genomic DNA as template. From top to bottom, the primer pairs indicated to the left of each gel image was used to confirm correct 5' integration junctions, correct 3' integration junctions, and the loss of the WT *PpDEKL* locus. Expected product sizes for each reaction are indicated within parentheses. (C) Results from genotyping PCR's with cDNA prepared from protonemal RNA as template. In the upper panel, the primers SS556 and SS557 were used to confirm the loss of *PpDEKL* transcripts. In the bottom panel, the Ubiquitin primers SS98 and SS99 were used to verify template quality. Expected product sizes for each reaction are indicated within parentheses.

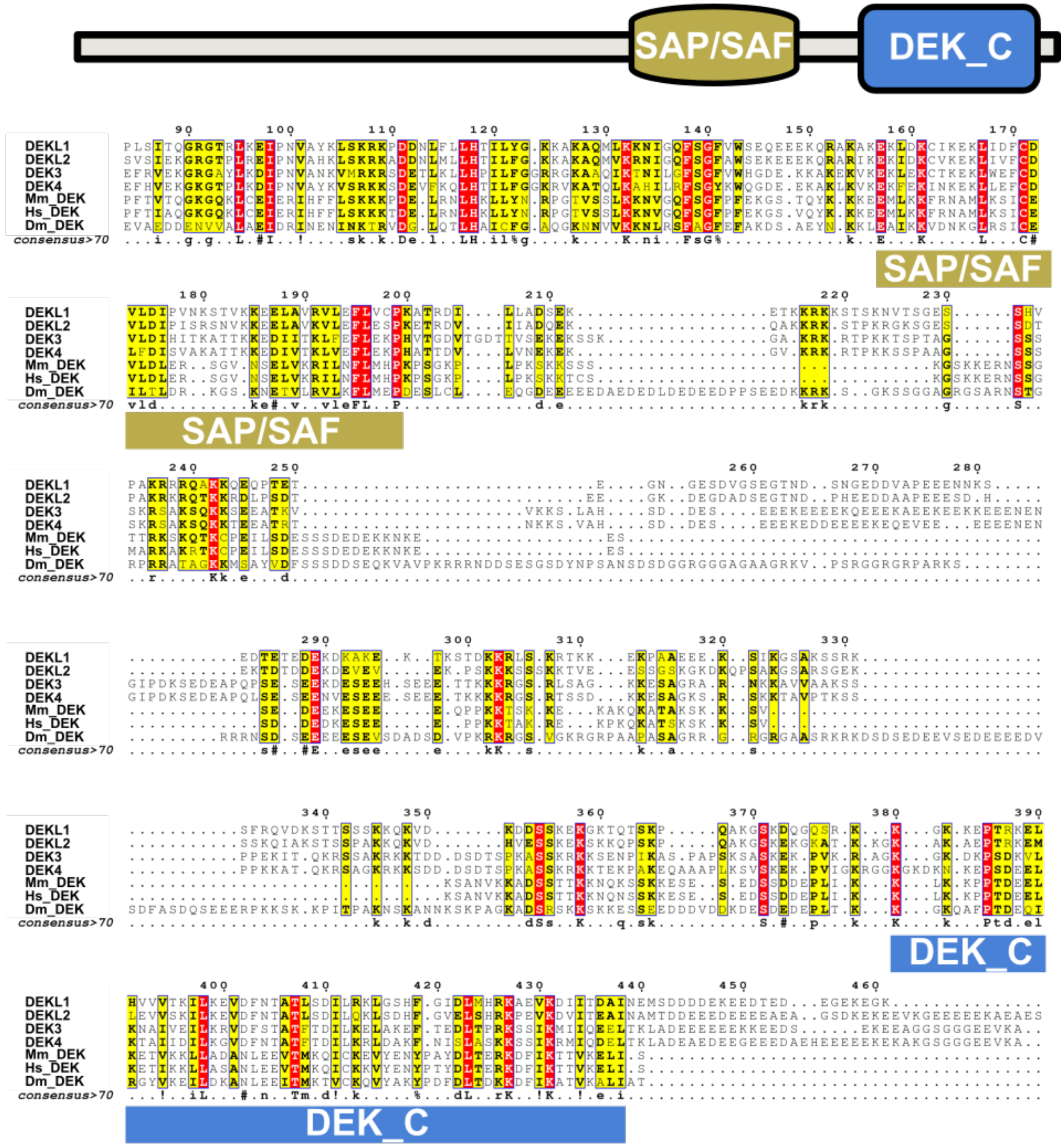

Fig. S2 DEK proteins are conserved between animals and plants

Alignment of Arabidopsis DEKL1 (AT3G48710), DEKL2 (AT5G63550), DEK3 (AT4G26630), DEK4 (AT5G55660) and human (Hs), mouse (Mm) and fruit fly (Dm) DEK amino acids sequences. Conserved domains are colored in red. %, !, and # in consensus symbols indicate amino acids FY, IV, and NDQEBZ, respectively.

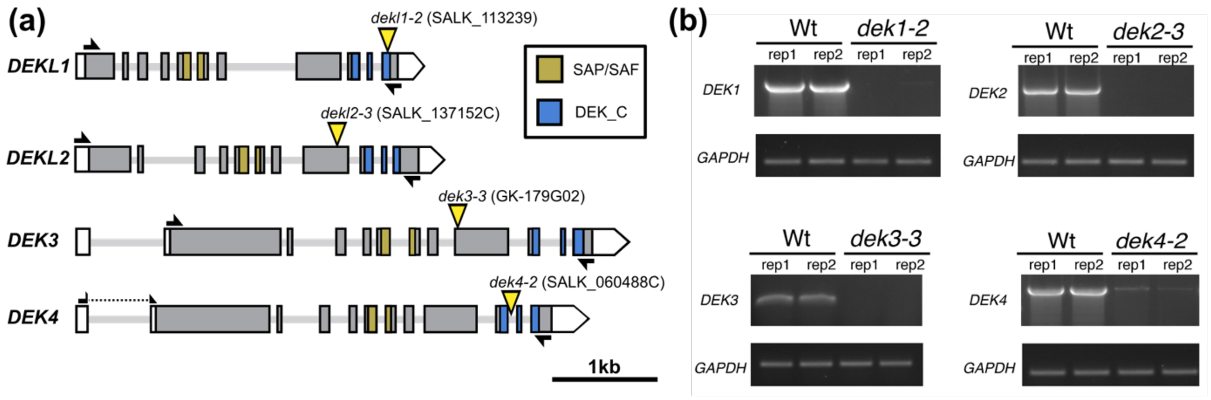

Fig. S3 *dek* T-DNA mutants examined in this study

(A) Schematic diagram of Arabidopsis 4 *DEK* genes. Conserved domains (SAP/SAF and DEK_C) are shown in color. T-DNA insertion sites are indicated by yellow triangles. Arrows indicates the positions of PCR primers for amplifying full-length transcripts. (B) Amplicon detection of *DEK* full-length transcripts in an agarose gel. *DEK* transcripts were amplified by RT-PCR in each *dek* mutant.

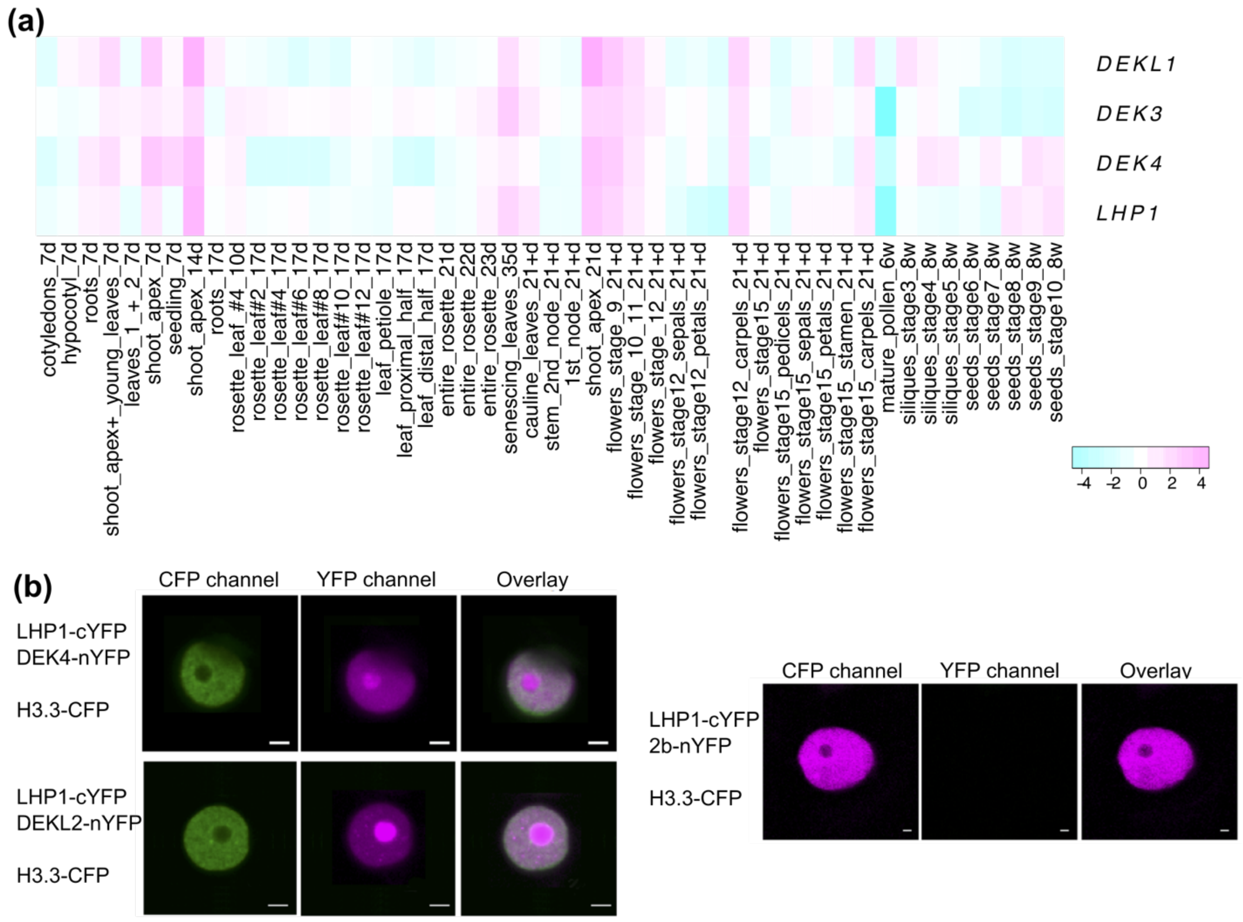

Fig. S4 *DEKs* and *LHP1* coexpress and their proteins interact in nuclei

(A) Heatmap of developmental expression patterns of *DEK* genes and *LHP1* based on the AtGenExpress dataset. *DEKL2* is not included in the original dataset. Expression values were scaled in each row. Microarray signals were normalized using the gcRMA algorithm (Wu et al. 2004). For each developmental stage, the values shown are the average across replicates. (B) DEKL2 and DEK4 interact with LHP1 *in vivo*. DEKL2 and DEK4 bind to LHP1 in the nucleus. DEKL2-nYFP or DEK4-nYFP or 2bnYFP were co-expressed with LHP1-cYFP and H3.3-CFP in *N. benthamiana* leaves under the control of the 35S promoter. The CMV 2b nuclear protein was used as a negative control. H3.3-CFP was used as a nuclear marker. Water-mounted sections of leaf tissue were examined by confocal microscopy. In the left top and bottom panels, the CFP and YFP channels are shown in green and magenta, respectively. In the right panel, the CFP channel is in magenta as a negative control. The scale bar is 2 μm.

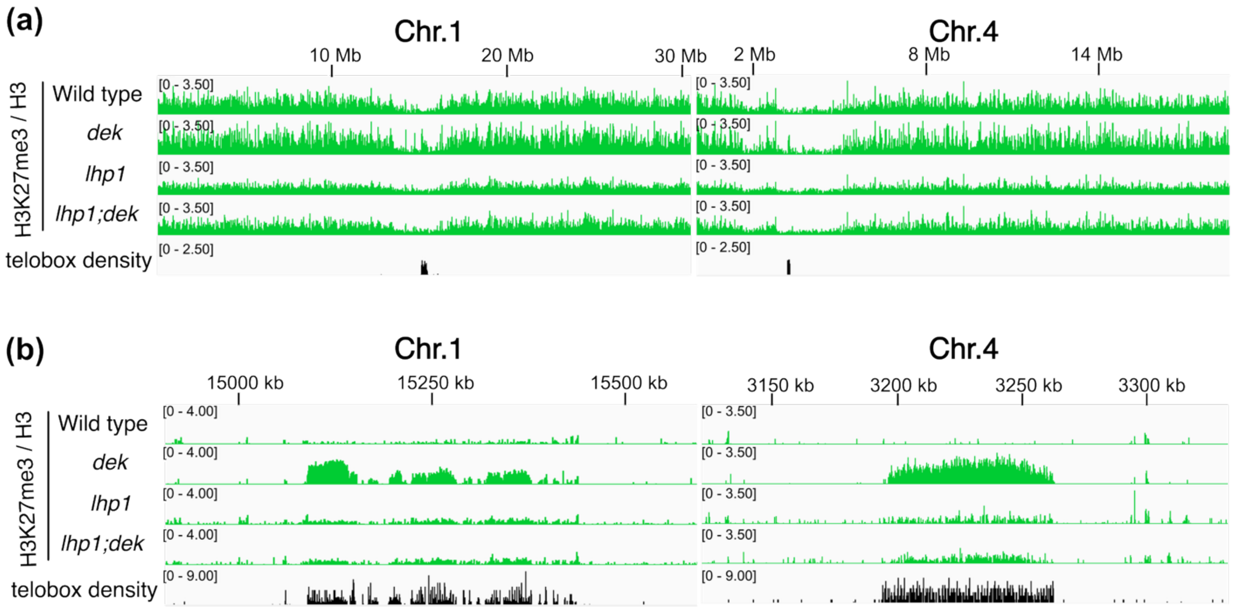

Fig. S5 Extended and detailed views of the H3K27me3 distribution at ITR genomic region.

Genome browser views of the ITR loci at two magnifications. (A) Entire chromosome landscapes for chromosomes 1 and 4. (B) Detailed views of ITR regions at higher magnification. Tracks show H3K27me3 signals normalized by H3 in each genotype and telobox motif density.

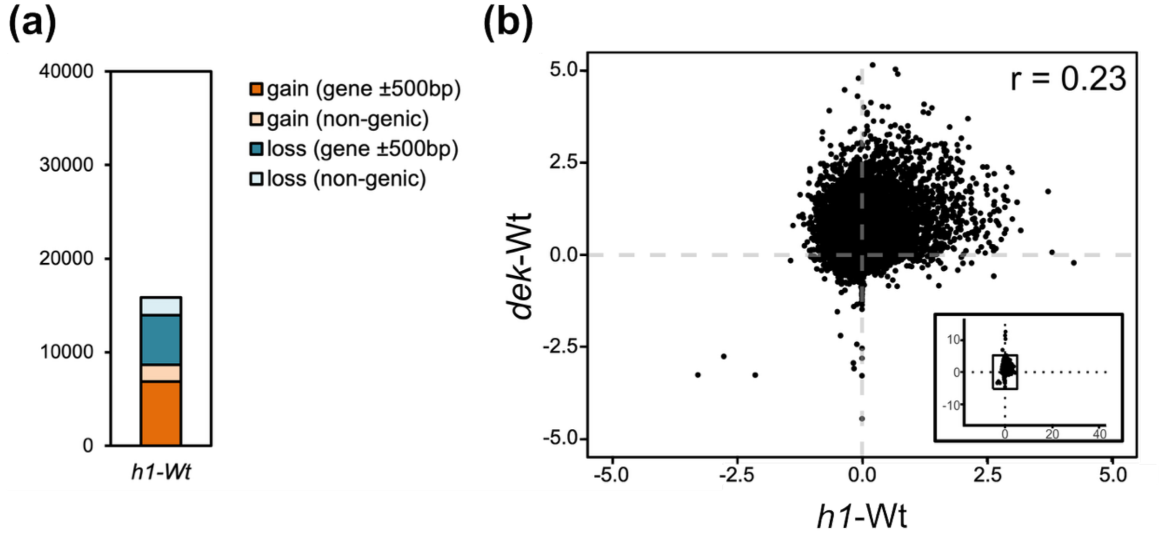

Fig. S6 Comparison of H3K27me3 changes in the *dek* and the *h1* mutants

(A) H3K27me3 gain and loss per-1kb bin (sliding window of 500 bp) between the histone *h1* double mutant and wild-type genotypes. Normalized coverage differences greater than 0.5 and less than -0.5 were considered gains and losses, respectively. Due to the 500 bp sliding window approach, some regions may be counted more than once. (B) Correlation of H3K27me3 changes in each mutant. Each dot indicates a single gene. The main plot excludes outliers; the inset displays all data. H3K27me3 differences are shown comparing *h1* double mutant vs wild-type and *dek* mutant vs wild type.

Table S1 **Primer sequences used for genotyping RT-qPCR, ChIP, and semi-quantitative for DEK transcripts and plasmid construction**

Experiments	Allele names	Stock name	AGI identifiers	Oligo names	Sequences (5′ - 3′)	Detection targets
Genotyping	*lhp1-6*	SALK_011762	AT5G17690	LH272	CCTTTCACAACCGAGTTTAT	genomic fragment
				LH291	CATTGGTGTGAGAAAGTGGCTGCTTTAG	genomic fragment
				LH2311	TGGTTCACGTAGTGGGCCATCG	T-DNA-genomic fragment fusion
				LH157	CGGTGGAAACAGTCGGAGAAA	T-DNA-genomic fragment fusion
	dekl1-2	SALK_113239	AT3G48710	LH1394	CTAGTCCTTTCCCTCTTTCTCACCT	genomic fragment
				LH1436	TGGTGACCAAAATTCTGAAGG	genomic fragment
				LH2311	TGGTTCACGTAGTGGGCCATCG	T-DNA-genomic fragment fusion
				LH1436	TGGTGACCAAAATTCTGAAGG	T-DNA-genomic fragment fusion
	dekl2-3	GK-288B07	AT5G63550	LH1440	TTGGTTGGATTACCGTCATTC	genomic fragment
				LH1441	ATTGCATTTATGGCCTCTGTG	genomic fragment
				LH1441	ATTGCATTTATGGCCTCTGTG	T-DNA-genomic fragment fusion
				LH2311	TGGTTCACGTAGTGGGCCATCG	T-DNA-genomic fragment fusion
	dek3-3	GK-179G02	AT4G26630	LH4391	TGGTCAACCCGTCAATGTTTCT	genomic fragment
				LH4561	GTGCGATGTGATCAGAAAGGAG	genomic fragment
				LH1162	ATATTGACCATCATACTCATTGC	T-DNA-genomic fragment fusion
				LH1389	AAAACAGGATAATGACCAAACCTG	T-DNA-genomic fragment fusion
	dek4-2	SALK_060488C	AT5G55660	LH1431	GCAGTCCCTACGAAATCCAGT	genomic fragment
				LH1447	AGCTTTAACCTCCTCTCCACCAC	genomic fragment
				LH1447	AGCTTTAACCTCCTCTCCACCAC	T-DNA-genomic fragment fusion
				LH2311	TGGTTCACGTAGTGGGCCATCG	T-DNA-genomic fragment fusion
	top1α-10	SALK_013164	AT5G55300	LH4545	CGATAGAAACCATCGAAGCA	genomic fragment
				LH4578	TTAACTGTGGAACCATTCTGATG	genomic fragment
				LH4545	CGATAGAAACCATCGAAGCA	T-DNA-genomic fragment fusion
				LH2311	TGGTTCACGTAGTGGGCCATCG	T-DNA-genomic fragment fusion
	atrx-3	SALK_024609	AT1G08600	LH4657	CATGGATATCTCTTGTTTACTTGGC	genomic fragment
				LH4646	CGACCAGTCAAGCTCAGATG	genomic fragment

			LH2311	TGGTTCACGTAGTGGGCCATCG	T-DNA-genomic fragment fusion
			LH4645	GAGGAATTAGCACAAGCACTCC	T-DNA-genomic fragment fusion

Experiments	Gene alias	AGI identifiers	Oligo names	Sequences (5′ - 3′)
RT-qPCR	*PP2a*	AT1G13320	LH2474	ATTCCGATAGTCGACCAAGC
			LH2475	AACATCAACATCTGGGTCTTCA
	FT	AT1G65480	LH0312	GGTGGAGAAGACCTCAGGAA
			LH0313	GGTTGCTAGGACTTGGAACATC
	SOC1	AT2G45660	LH1741	AGCAGCTCAAGCAAAAGGAG
			LH1742	TTGACCAAACTTCGCTTTCA
	PI	AT5G20240	LH2322	CGCCATCATCTTCTCATTTCT
			LH2323	AACATGGCCTCGACAAAGTC
	SEP3	AT1G24260	LH1605	TTGAAGGCACATTGGGTTCT
			LH1606	GAAAGCTGTACGAGTTTTGCAG
	AT1G80160	AT1G80160	LH1571	GATAATCACATTTCTTTCCAGTGC
			LH1572	TCAACCACTGCTCTCACATACTC
	FLC	AT5G10140	LH304	GACTGCCCTCTCCGTGACTA
			LH305	TTCTCAACAAGCTTCAACATGAG
	MAF1	AT1G77080	LH1720	ACTGCTCTGTCCGTAAGTAGAGC
			LH1721	TCTCCTTTTCTTTAAGGGACTCG
	MAF2	AT5G65050	LH536	ATGATGGGGGAAGTGAAGTCC
			LH537	CCATGACATTCCTCTGTCACC
	MAF3	AT5G65060	LH827	TTACTTGAGCAGCGAAAGAGTC
			LH2909	GAAAGGGAGAAGTTGCTGATAGAAGAG
	MAF4	AT5G65070	LH2911	TGAAGACCCATCAAGAGAAGG
			LH2912	GACTTCTTCATCTTCCCCATCTT
	MAF5	AT5G65080	LH2917	GCTGAAAGAAAAGAACAAGGTTCTA
			LH2918	AGAGCTATTTTCCGGTGACATTAC
	ABI3	AT3G24650	LH3311	TCAGTTACCTACCATGGACC
			LH3312	TTTCTGGTTTCCATCCCTGC
	AG	AT4G18960	PW232	ATCCGATCCAAGAAGAATGAG
			PW233	TTTCAGCTATCTTTGCACGA
	AP3	AT3G54340	LH4336	AGCGCAAGTTCAAATCTCTTG
			LH4337	AGGATCTTCAGCTCTTAGTTCCAG
	UBQ10	AT4G05320	LH2903	TTCTCTCAATTCTCTCTACCGT
			LH2904	TGGCCTTAACGTTGTCGA
	LFY	AT5G61850	LH4380	ATCGCTTGTCGTCATGGCTG
			LH4381	GCAACCGCATTGTTCCGCTC
	AP1	AT1G69120	LH4334	GCAGCACCAAATCCAGCATC
			LH4335	ATGGAAATGCTTCATGCGGC

Experiments	Gene alias	AGI identifiers	Oligo names	Sequences (5′ - 3′)
Semi quantitative RT-PCR	*DEKL1*	AT3G48710	LH1393	ATGGCGACTGAAACCCTAGAATTG
			LH1394	CTAGTCCTTTCCCTCTTTCTCACCT
	DEKL2	AT5G63550	LH1531	CGTTGAATTAGGGTTTTACAATTTCGAG
			LH1449	ATCCTTTGGCTCTTCTTTTTCCTTCTC
	DEK3	AT4G26630	LH1396	TTAGGCTTTCACCTCCTCACCA
			LH1444	caccATGGGGGAAGATACAAAGGC
	DEK4	AT5G55660	LH1880	AAGCCTTTGGATAGCGAAGC
			LH1447	AGCTTTAACCTCCTCTCCACCAC
	GAPDH	AT3G26650	LH0675	AATGCGAAGCCTGCTTGA
			LH0676	AATGAAAGGCCCAAAAATCTAA
ChIP-qPCR	*AP3*	AT3G54340	LH4790	ACAACCATCGATGTCCGTTGA

			LH4791	AGAAGTAAAGGGTCCACTTGAGT
	PI	AT5G20240	LH4796	TCAATCACATGCAAAGAGTGTTC
			LH4797	ACAGTCATGGAAGGCTTGCT
	AG	AT4G18960	MD032	CTAATCAAATTTTGCCCTAAACG
			MD033	TCCTAGCTCCGATTGGTACG
Construction for Bi-FC	*DEKL2*	AT5G63550	LH1448	CACCATGGCGACTGAAACCCTAGAT
			LH1449	ATCCTTTGGCTCTTCTTTTTCCTTCTC
	DEK4	AT5G55660	LH1446	CACCATGGGGGAAGAAGATACAAAAG
			LH1447	AGCTTTAACCTCCTCTCCACCAC
	H3.3	AT4G40030	LH0565	CACCATGGCTCGTACCAAGCAAAC
			LH1234	AGCGCGTTCACCTCTGATAC
Construction for moss KO line	*PpDEKL*		PpDEKL-5’-F	caaatggatccCTCCTACCACCACCACCACC
			PpDEKL-5’-R	caaatggatccATCTCGACCTGGACAAGAGTGG
			PpDEKL-3’-F	caaatgttaacTCACTGATGTTTATGTCAGGAAGG
			PpDEKL-3’-R	caaatgttaacTTTCCATCTACTTTTGCCGCATGG
Expression in Moss	*Ubiquitin*		SS98	ACTACCCTGAAGTTGTATAGTTCGG
			SS99	CAAGTCACATTACTTCGCTGTCTAG
	PpDEKL		SS556	CGACGATGCAGATAGCGACAACTC
			SS557	ACTCACCGCCTTTCACAATAGACTTC
Moss genotyping	*PpDEKL knock out 5'*		LH3919	TTTGGCCCCCTTCCTAATGG
			SS188	AGTCTTTACGGCGAGTTCTGTTAGGT
	PpDEKL knock out 3'		SS191	AGGCATGCCCGCTGAAATC
			SS558	CAGCAATGGACCTAGTTCCGTCTG
	PpDEKL genomic		SS556	CGACGATGCAGATAGCGACAACTC
			SS557	ACTCACCGCCTTTCACAATAGACTTC

Table S2 Gene Ontology enrichment and adjusted *p*-values of genes with high loadings on Principal component 1 (PC1)

Category	Go term ID	Description	Enrichment	Adjusted *p* value
Biological Process	GO:0045944	positive regulation of transcription by RNA polymerase II	7.62	9.38E-13
	GO:0042221	response to chemical	1.79	4.81E-11
	GO:0009753	response to jasmonic acid	4.86	3.38E-09
	GO:0050896	response to stimulus	1.40	3.50E-09
	GO:0048367	shoot system development	2.39	3.87E-08
	GO:0006351	DNA-templated transcription	1.71	1.17E-06
	GO:0010022	meristem determinacy	16.46	4.71E-06
	GO:0032501	multicellular organismal process	1.49	2.56E-04
	GO:0022414	reproductive process	1.69	4.09E-04
	GO:0065007	biological regulation	1.28	8.95E-04

	GO:0050793	regulation of developmental process	2.10	1.10E-03
	GO:0032502	developmental process	1.41	1.95E-03
	GO:0009718	anthocyanin-containing compound biosynthetic process	9.16	3.76E-03
	GO:0034758	positive regulation of iron ion transport	43.20	7.35E-03
	GO:0016102	diterpenoid biosynthetic process	5.80	8.84E-03
	GO:2000241	regulation of reproductive process	2.95	1.14E-02
	GO:0044419	biological process involved in interspecies interaction between organisms	1.63	1.34E-02
	GO:0009611	response to wounding	3.02	1.42E-02
	GO:0051239	regulation of multicellular organismal process	2.30	2.05E-02
	GO:0009607	response to biotic stimulus	1.60	2.99E-02
	GO:0051707	response to other organism	1.60	3.53E-02
Cellular Component	GO:0005576	extracellular region	2.52	4.05E-04
	GO:0110165	cellular anatomical entity	1.47	3.32E-02
Molecular Function	GO:0000977	RNA polymerase II transcription regulatory region sequence-specific DNA binding	16.63	1.62E-20
	GO:0003700	DNA-binding transcription factor activity	2.66	3.07E-11
	GO:0140110	transcription regulator activity	2.57	1.12E-10
	GO:0046983	protein dimerization activity	3.23	1.28E-07
	GO:0001067	transcription regulatory region nucleic acid binding	2.64	8.55E-05
	GO:0005506	iron ion binding	3.33	9.62E-04
	GO:0016705	oxidoreductase activity, acting on paired donors, with incorporation or reduction of molecular oxygen	2.97	4.06E-03
	GO:0016838	carbon-oxygen lyase activity, acting on phosphates	8.96	6.19E-03
	GO:0009975	cyclase activity	12.19	2.05E-02

Reference:

Wu Z, Irizarry RA, Gentleman R, Martinez-Murillo F, and Spencer F. A Model-Based Background Adjustment for Oligonucleotide Expression Arrays. Journal of the American Statistical Association. 2004:**99**(468):909–917. https://doi.org/10.1198/016214504000000683
